# Modification of mesenchymal stem cells by HMGB1 promotes the activity of Cav3.2 T-type calcium channel via PKA/β-catenin/γ-cystathionase pathway

**DOI:** 10.1186/s13287-021-02677-z

**Published:** 2022-01-10

**Authors:** Hao Wu, Xiaodong Xie, Mingyang Sun, Min Chen, Xuan Tao, Xin Fang, Xiaohu Meng, Wei Wei, Min Yu

**Affiliations:** 1 Division of General Surgery, The Second Affiliated Hospital of Nanjing Medical University, Nanjing, China; 2 Department of Gastroenterology, Nanjing University Medical School Affiliated Nanjing Drum Tower Hospital, Nanjing, China; 3 Department of Vascular Surgery, Affiliated Hangzhou First People’s Hospital, Zhejiang University School of Medicine, Hangzhou, China; 4 Department of Anesthesiology and Perioperative Medicine, The First Affiliated Hospital of Nanjing Medical University, Nanjing, China

**Keywords:** Mesenchymal stem cell, High mobility group box 1, Cav3.2 T-type calcium channel, Cav3.2 T type calcium channel, Protein kinase A, β-Catenin, γ-Cystathionase, Glycogen synthase kinase 3β, Cyclic adenosine monophosphate

## Abstract

**Background:**

Mesenchymal stem cells (MSC) hold great promise for treating cardiovascular disease. Recently, we genetically modified MSCs with high mobility group box 1 (HMGB1), and these cells demonstrated high mobility by efficient migrating and homing to target neointima. The possible mechanism was investigated in the current study.

**Methods:**

Rat MSCs were transfected with lentivirus containing HMGB1 cDNA to yield MSC-H cell line stably overexpressing HMGB1. The MSC-C cells which were transfected with empty lentivirus served as negative control, and the differentially expressed genes were analyzed by microarray. The cell mobility was determined by transwell migration assay. Intracellular free calcium and the expression of Cav3.2 T-type calcium channel (CACNA1H) were assayed to analyze activity of CACNA1H-mediated calcium influx. H_2_S production and γ-cystathionase expression were examined to assess the activity of γ-cystathionase/H_2_S signaling. The interaction of HMGB1 with γ-cystathionase in MSC-H cells was analyzed by co-immunoprecipitation. Luciferase reporter assay was performed to determine whether the promoter activity of γ-cystathionase was regulated by interaction of β-catenin and TCF/LEF binding site. Intercellular cAMP, PKA activity, phosphorylation of β-catenin, and GSK3β were investigated to reveal cAMP/PKA mediated β-catenin activation.

**Result:**

Microarray analysis revealed that differentially expressed genes were enriched in cAMP signaling and calcium signaling. CACNA1H was upregulated to increase intracellular free calcium and MSC-H cell migration. Blockage of CACNA1H by ABT-639 significantly reduced intracellular free calcium and cell migration. The γ-cystathionase/H_2_S signaling was responsible for CACNA1H activation. H_2_S production was increased with high expression of γ-cystathionase in MSC-H cells, which was blocked by γ-cystathionase inhibitor DL-propargylglycine. Upregulation of γ-cystathionase was not attributed to interaction with HMGB1 overexpressed in MSC-H cells although γ-cystathionase was suggested to co-immunoprecipitate with oxidized HMGB1. Bioinformatics analysis identified a conserved TCF/LEF binding site in the promoter of γ-cystathionase gene. Luciferase reporter assay confirmed that the promoter had positive response to β-catenin which was activated in MSC-H cells. Finally, cAMP/PKA was activated to phosphorylate β-catenin at Ser657 and GSK3β, enabling persisting activation of Wnt/β-catenin signaling in MSC-H cells.

**Conclusion:**

Our study revealed that modification of MSCs with HMGB1 promoted CACNA1H-mediated calcium influx via PKA/β-catenin/γ-cystathionase pathway. This was a plausible mechanism for high mobility of MSC-H cell line.

**Supplementary Information:**

The online version contains supplementary material available at 10.1186/s13287-021-02677-z.

## Background

Mesenchymal stem cells (MSC) are non-hematopoietic pluripotent stem cells. They have the potential of differentiation into a variety of adult tissue cells and secrete a variety of biologically active substances, enabling their application in tissue repair and regeneration [1]. Previous studies have shown that transplantation of MSCs restore physiological function of endothelium and reduce neointima formation [2]. On the one hand, they differentiate into a new generation of endothelial cells to replace loss of host endothelial cells. On the other hand, they release anti-inflammatory factors and growth factors to create a nourishing microenvironment for vascular regeneration. However, MSC therapy still encounters many problems in clinical application, such as low homing efficiency [3] and fear of tumorigenesis [4]. Currently, many studies focus on how to manipulate MSC to improve therapeutic effects.

Recently, we promoted the mobility of MSCs by lentiviral transfection of high mobility group box 1 (HMGB1) and generated MSC-H cell line stably overexpressing HMGB1 [5, 6]. HMGB1 is a damage associated molecular pattern and normally exists in the nucleus binding to DNA and regulating gene transcription. The danger signals stimulate extracellular release of HMGB1 to induce inflammation and immune response [7]. With in-depth research on physiological function of HMGB1 other than proinflammatory property, HMGB1 has been discovered to promote tissue repair by stimulating the migration and differentiation of stem cells [8]. The reason why HMGB1 plays distinct biological roles is closely linked to the redox state of HMGB1. HMGB1 has three cysteine residues with two located at position 23, 45 in A-box and one at position 106 in B-box. When cysteine residues at position 23 and 45 are oxidized to form disulfide bond, oxidized HMGB1 induces proinflammatory response. If three cysteine residues are all oxidized, HMGB1 loses biological activity. In comparison, reduced HMGB1 is physiologically favorable and primes stem cells for proliferation and migration. The stem cells treated with reduced HMGB1 sustain increase in cell cycling by transition of quiescent stem cells from *G*_0_ to *G*_Alert_, facilitating a quick response to tissue injury [8]. Our study found that MSC-H cells not only migrated increasingly to the neointima but also acquired the great potential of endothelial differentiation, thereby inhibiting the neointimal formation during pathogenesis of vascular disease [5, 6]. Such effects were attributed to the expression and extracellular release of HMGB1 by MSC-H cells. The HMGB1 content was significantly increased in the cytoplasm and culture medium of MSC-H cells, although there was no obvious change of HMGB1 level in the nucleus. Neutralizing extracellular HMGB1 by specific antibody effectively blocked the migration and endothelial differentiation of MSC-H cells, suggesting MSC-H cells were stimulated by self-secreted HMGB1 in an autocrine manner [6]. Although MSC-H cells had the superior property of promoting vascular regeneration, the underlying mechanism was not yet intensively investigated. The current study was to unravel signaling pathways for high mobility of MSC-H cells.

## Materials and methods

### Chemicals and reagents

All chemicals and reagents were purchased from Sigma unless otherwise stated.

### Cell culture and transfection

Rat MSCs were purchased from Cyagen Biosciences China and grown in DMEM supplemented with 10% Gibco fetal bovine serum. The cells were cultured at 37 °C in a humidified atmosphere of 5% CO_2_. The MSC-H cells stably overexpressing HMGB1 were constructed by transfection with a recombinant lentivirus (sequence elements: Ubi-MCS-FLAG-SV40-puromycin) containing HMGB1 cDNA. The empty vector was transfected into MSC-C as the negative control. The experiment of HMGB1 knock-down was conducted by transfecting MSC-H cells with three plasmids (sequence elements: hU6-MCS-CMV-Neomycin) carrying shRNAs targeting HMGB1. The cells were labeled by siH1, siH2, and siH3, respectively, and siC referred to the cells transfected with the empty plasmid. All lentivirus and plasmids were designed and produced from Genechem China. The transfection followed the procedures as previously described, and the efficiency was verified by Western blot [5].

### RNA microarray

Microarray analysis of gene expression was performed by Genechem (Shanghai, China). Briefly, total RNA was extracted from MSC-H and MSC-C cells by using RNAeasy™ plus animal RNA isolation kit with spin column (Beyotime, China). RNA quality and concentration were assessed by using RNA 6000 Nano kit on Agilent 2100 bioanalyzer. RNA samples were then amplified by performing reverse transcription to obtain cDNA, followed by T7 in vitro transcription to produce complementary RNA. After RNA purification and quantification, the complementary RNA was converted into single-stranded complementary DNA. The RNA residue in the samples was hydrolyzed by RNase H. The single-stranded DNA was purified, fragmented, and biotin-labeled for hybridization to rat Clariom™ S arrays (Thermo Fisher Scientific, USA). The hybridized arrays were stained and washed by Affymetrix GeneChip Fluidics Station 450. Image signals were scanned by Affymetrix GeneChip Scanner 3000 and transformed to digital data by Affymetrix GeneChip Command Console software. The data files were transferred to the Affymetrix GeneChip Expression Console software for analysis of gene expression patterns. The cut-off value for upregulated and downregulated genes was set as an absolute fold change of > 1.5 with a *P* value < 0.05. The GO and KEGG pathway enrichment analysis of differentially expressed genes were performed to determine the crucial GO terms and signaling pathways associated with HMGB1 modification.

### Transwell migration assay

The cell mobility was evaluated by transwell assay. Each well of Corning 24-well plate was separated to the upper and lower compartment by inserting a chamber with 8.0-µm-pore filter membrane on the bottom. The upper compartment was added with 1 × 10^5^ cells suspended in serum-free DMEM, and the lower compartment was filled with DMEM with 15% fetal bovine serum as attractant. After being cultured at 37 °C and 5% CO_2_ for 24 h, the cells that migrated through the filter and adhered to the lower side of membrane were fixed in methanol, stained with 0.1% crystal violet solution, and visualized by phase-contrast microscopy.

### Subcellular fractionation

The cytoplasmic fraction from cultured cells were obtained as previously described [9]. Briefly, cells were suspended in cold cytoplasm lysis buffer (10 mM TrisHCl pH 8.0, 60 mM KCl, 1 mM EDTA, protease inhibitor and 0.5% NP-40) and gently triturated using a 26-gauge needle. The cell lysate was centrifuged at 1000 × *g* for 5 min at 4 °C, and the supernatant was collected as the cytoplasmic fraction.

### Western blot

Protein samples were separated by SDS-PAGE, transferred to polyvinylidene difluoride membranes, and blocked with 5% nonfat milk solution. The membranes were cut into strips according to molecular weight of target protein, and each strip was incubated separately with primary antibody for target protein overnight followed by corresponding secondary antibody for 1 h. Primary antibodies to HMGB1, Lamin-B1, Tubulin, and CACNA1H were purchased from Abcam while other antibodies were obtained from Cell Signaling Technology. Antibody binding was visualized using the BeyoECL Star system (Beyotime, China).

### Calcium imaging

The changes of intracellular free calcium were detected by Rhod-3 calcium imaging (Thermo Fisher, USA). The adherent cells were washed with phosphate buffer before being incubated with loading buffer containing 10 μM Rhod-3 dye in dark at room temperature for 30 min. The cells were washed with phosphate buffer again to remove the residue of loading buffer. Then incubation buffer containing 2.5 mM probenecid was added to the cells to reduce the baseline signal. The cells were incubated in dark at room temperature for 30 min before the incubation buffer was removed. Finally, nuclei were contra-stained with DAPI for 2 min, and the cells were ready for fluorescent microscopy. The intracellular free calcium was quantified by red fluorescence intensity.

### H_2_S measurement

The extracellular H_2_S concentration was measured by the colorimetric method (Solarbio, China). Briefly, the cells were cultured in phenol red-free DMEM supplemented with 10% fetal bovine serum for 48 h. The supernatants were collected and added with the solution of zinc acetate, which reacted with H_2_S produced in the culture to form zinc sulfide. Next, hydrochloride acid solution of *N*,*N*-dimethyl-p-phenylenediamine sulfate was added to dissolve zinc sulfide. In the presence of ammonium ferric sulfate, the reaction yielded methylene blue with an optic absorption peak at 665 nm. The absorbance was measured at 665 nm by a spectrophotometer. H_2_S was calculated against a calibration curve of NaHS and normalized by the protein concentration determined by Bradford protein assay (Beyotime, China). Intracellular H_2_S level was measured by WSP-5 fluorescent probes (Cayman Chemical, USA). Briefly, the adherent cells were washed and incubated with 50 μM WSP-5 in dark at 37 °C for 20 min. WSP-5 selectively and rapidly reacted with H_2_S in live cells to release a fluorophore that displayed excitation/emission peaks of 502/525 nm. The fluorescent intensity was observed by a fluorescent microscope.

### γ-cystathionase (CTH) activity assay

The activity of CTH was determined by the efficiency of catalyzing an α,γ-elimination reaction of L-cystathionine to produce α-ketobutyrate [10, 11]. Briefly, 50 µL cell lysate was mixed with 500 µL loading buffer which was prepared from 25 µL 1.3 mM pyridoxal phosphate, 25 µL 13 mM EDTA, 250 µL 45 mM cystathionine, and 275 µL of 0.1 M pH 7.5 phosphate buffer containing 0.05 mM 2-mercaptoethanol. The mixture was incubated at 37℃ for 15 min. The reaction was stopped by adding 100 µL 10% perchloric acid. After centrifugation at 1500 × *g* for 10 min, 25 µL of supernatant was transferred to 625 µL 0.194 mM NADH solution and kept at 37 °C. The sample was ready for spectrophotometry. The absorbance was measured at 340 nm now, continuing for 3 min after 10 IU L-lactic dehydrogenase were added. The difference between the absorbance before and after adding lactic dehydrogenase corresponded to the amount of α-ketobutyrate formed in the course of the CTH catalytic reaction. The control samples without adding cystathionine were prepared and measured in the same way as the examined samples. The CTH activity was expressed as nmoles of α-ketobutyrate formed during 1 min incubation at 37 °C/1 mg of protein.

### Co-immunoprecipitation

Co-immunoprecipitation for identifying the interaction of HMGB1 and CTH was performed according to the procedures previously described [9, 12]. Briefly, protein A agarose beads (Roche, USA) were washed twice with phosphate buffer, and then prepared at 50% concentration with phosphate buffer. 100 ul of protein A agarose beads were loaded to each 4 mg of total protein samples, and incubated with agitation for 10 min at 4 °C to remove non-specific impurity protein. The samples were centrifugated at 14,000 × *g* for 15 min at 4 °C, and the supernatant was transferred to a new centrifuge tube. The protein concentration was determined by Bradford method. The total protein was diluted to 2 ug/ul with phosphate buffer to reduce the detergent concentration in the lysates. For immunoprecipitation, 5ul rabbit anti-HMGB1 antibody (Abcam, USA) was mixed with 500 ul total protein and incubated overnight at 4 °C to allow antigen–antibody binding. The antigen–antibody complexes were captured by incubation with 100 ul protein A agarose beads overnight at 4 °C. After centrifugation at 14,000 × *g* for 10 s, the antigen–antibody-agarose bead complexes were collected and washed 3 times with pre-chilled phosphate buffer. The antigen–antibody-agarose bead complex was resuspended with 60 ul 2 × SDS loading buffer, boiled for 5 min to dissociate antigen–antibody complexes from beads. The beads were pelleted by centrifugation, and the supernatant was boiled again for 5 min before Western blot analysis.

### Protein thiol assay

Firstly, HMGB1 of all isoforms was isolated and purified from the cell lysates by immunoprecipitation. HMGB1 produced from MSC-H carried flag-tags, making it readily purified by FLAG immunoprecipitation kit (Sigma, USA). The cells were washed twice with phosphate buffer and lysed by a pre-chilled mixture of 20 mM Tris(pH7.5), 150 mM NaCl, 1% Triton X-100, and proteinase inhibitors (Beyotime, China). The lysate was centrifugated at 14,000 × *g* for 10 min, and the supernatant was collected for immunoprecipitation with anti-flag M2 affinity gel (FLAGIPT1, Millipore, USA). The gel beads were selective for recombinant HMGB1 with flag-tags and exhibited low non-specific binding of other proteins. After washing with phosphate buffer, the bound protein was competitively displaced from the gel with the elution solution of 3 × flag peptide. HMGB1 antibody was used for the immunoblotting of target protein in order to verify that HMGB1 was purified for protein thiol assay. Then the free thiol groups in HMGB1 were quantified by the colorimetric assay (Abcam, USA). 50 μg purified HMGB1 was diluted with assay buffer to the final volume of 100 μl and mixed with one vial of Thiol Blue sensor which reacted with free thiol groups. The mixture was incubated with agitation at room temperature for 1 h, followed by flowing through a spin column to remove the excess of Thiol Blue sensor. The flow-through solution was collected and diluted fivefold with assay buffer. The absorbance at 680 nm and 280 nm was measured by a microplate reader. The reduced HMGB1 (rHMGB1) was assigned to the non-oxidized control for the assay. The mixture of oxidized HMGB1 and rHMGB1 was prepared from incubation of rHMGB1 with 50 μM hydrogen peroxide in our previous study [13] and now served as the oxidized control group to determine whether HMGB1was oxidized in MSC-H cells. The amount of free thiol in HMGB1 was calculated with the formula provided by the manufacturer and normalized to the non-oxidized control.

### Dual luciferase assay

The 805 bp DNA fragment from − 686 to + 119 of rat CTH promoter (NM_017074) was cloned and inserted into GV238 (sequence elements: MCS-firefly-Luciferase) to construct wide-type luciferase reporter plasmid (pCTH-wt). The point mutation at + 33 base was introduced to eliminate the inference of ATG sequence from + 31 to + 33 on the luciferase transcription. A conserved TCF/LEF binding sequence 589 bp upstream of transcriptional start site was identified on CTH promoter by bioinformatics analysis. The mutation of TCF/LEF binding site was introduced into promoter plasmid to construct a pCTH mutant (pCTH-mut). A truncated CTH promoter sequenced from − 562 to + 119 was cloned into GV238. The construct (pCTH-tru) consisted of key transcription factor binding motifs with exception of TCF/LEF binding site. All plasmids were designed and constructed by Genechem (Shanghai, China). Dual-Glo luciferase assay (Promega, USA) was performed to assess CTH promoter activity in MSC-H cells. Briefly, the cells were transfected with promoter plasmids until grown to approximately 60% confluent in 24-well plates. The stimulation of β-catenin on CTH promoter activity was demonstrated by co-transfection of pCTH-wt and pCMV-S33Y-β-catenin into 293 T cells and verified in the experiment of Wnt/β-catenin inhibition by incubation of MSC-H with a concentration gradient of XAV939. pCMV-S33Y-β-catenin was a construct of oncogenic stable mutant form of β-catenin [14]. For the mock assay, the empty plasmid was transfected to the cells. The effect of β-catenin-TCF/LEF binding on promoter activity was determined by transfection of pCTH-tru and pCTH-mut to MSC-H cells. The activity of Firefly luciferase was assayed 48 h after transfection and normalized to that of Renilla luciferase reporter (sequence elements: TK promoter- Renilla_luciferase). The relative luciferase activity was expressed in percentage.

### cAMP measurement

Intracellular cAMP was measured by cAMP solid-phase sandwich ELISA (Invitrogen, USA). 1 × 10^6^ cells was lysed by 1 ml of 0.1 mol/l hydrogen chloride for 20 min at room temperature. The cell debris was pelleted and removed by centrifugation at 1000 × *g* for 10 min. 100 μl supernatant of each test sample was loaded to a 96-well plate together with 50 μl neutralizing reagent, 50 μl alkaline phosphatase-conjugated cAMP, and 50 μl rabbit anti-cAMP antibody and incubated with slow agitation at room temperature for 2 h. The plate was precoated with anti-rabbit antibody to capture rabbit anti-cAMP antibody in the mixture. The cAMP of test samples competed with alkaline phosphatase-conjugated cAMP for the limited amount of cAMP antibody bound to the plate. With the increase of cAMP levels in test samples, alkaline phosphatase-conjugated cAMP captured by the coating antibody was reduced and visualized by reacting with pNpp substrate. The absorbance at 405 nm was inversely proportional to the cAMP concentration in test samples. The cAMP standard was serially diluted and assayed as test samples to generate a standard curve. The cAMP concentration was normalized to total protein concentration and expressed as pmoles cAMP per mg of total protein.

### PKA activity assay

The PKA activity was measured by the colorimetric assay (Invitrogen, USA). 2 × 10^6^ cells were incubated on ice for 30 min with 100 μl Tris-based cell lysis buffer containing NP-40, protease inhibitor cocktail, PMSF and sodium orthovanadate. After centrifugation at 8000 × *g* for 10 min at 4 ℃, the supernatant was collected, diluted tenfold with 1 × kinase reaction buffer, and transferred to a 96-well plate at 40 μl per well. The PKA standard was diluted to a concentration gradient from 10 to 0.625 U/ml and loaded to the 96-well plate as test samples. The serial dilutions were prepared to generate a standard curve. 10 μl ATP working solution was added to each well, followed by incubation with agitation at 4 °C for 90 min to allow phosphorylation of immobilized PKA substrate bound to the microtiter plate. The reaction was stopped by aspirating the plate and washing with wash buffer. The mixture of rabbit anti-phospho PKA substrate antibody and horseradish peroxidase-conjugated anti-rabbit antibody were loaded at 50 μl per well and incubated for 60 min at room temperature to probe the phosphorylated substrate on the plate. The antibody binding was visualized by TMB chromogen solution. The absorbance was measured at 450 nm by a microplate reader and translated into PKA concentration by use of the standard curve. The PKA activity was obtained by multiplying PKA concentration by the total volume of test samples and finally being normalized to cell count.

### Statistical analysis

All experiments were repeated three to five times. Data were processed using GraphPad Prism 5 (GraphPad Software, Saint Diego, CA). Data were expressed as mean ± standard deviation and compared between groups by one-way ANOVA. A *P* value of < 0.05 was considered statistically significant.

## Results

### Microarray analysis of differentially expressed genes after upregulation of HMGB1

**Fig. 1:**
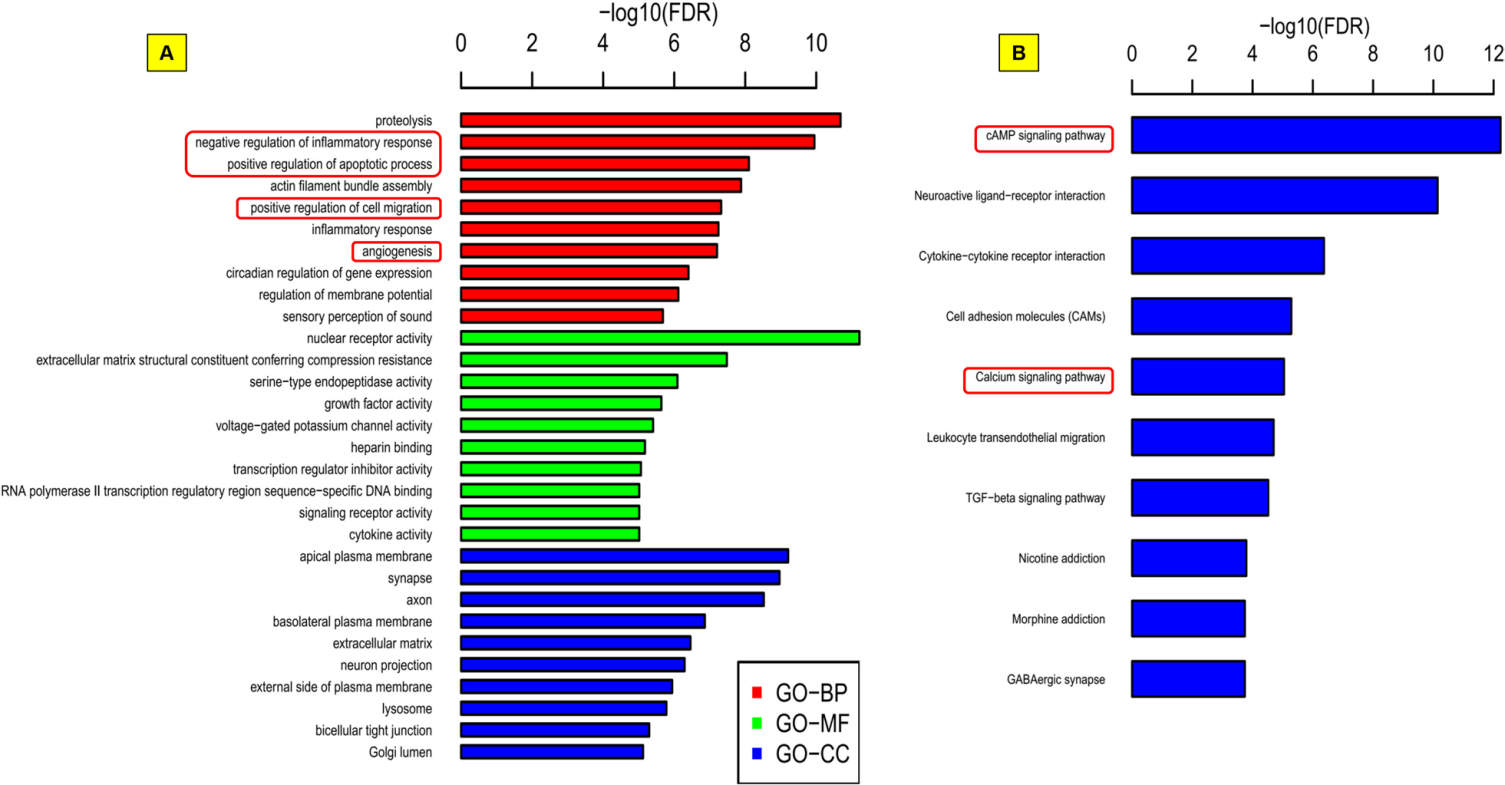
Microarray analysis of differentially expressed genes in rat mesenchymal stem cells after HMGB1 modification. The inclusion criteria for differentially expressed genes were the absolute fold change of RNA levels > 1.5 and *P* value < 0.05. The analysis yielded 963 (551 upregulated and 412 downregulated) genes. The majority of genes were enriched in HMGB1-mediated biological processes such as negative regulation of inflammatory response, positive regulation of apoptotic process, positive regulation of cell migration and angiogenesis (**a**). Notably, multiple key pathways were enriched including cAMP signaling and calcium signaling (**b**)

Differentially expressed genes between MSC-H and MSC-C cells were investigated by microarray to reveal the possible pathways that were involved after HMGB1 upregulation. The inclusion criteria for differentially expressed genes were the absolute fold change of RNA levels > 1.5 and *P* value < 0.05. Finally, 963 genes including 551 upregulated and 412 downregulated genes were identified by the analysis (Additional file 1). These genes were involved in HMGB1-mediated biological process such as negative regulation of inflammatory response, positive regulation of apoptotic process, positive regulation of cell migration, and angiogenesis (Fig. 1a). Furthermore, KEGG enrichment analysis revealed that the majority of genes were enriched in top five pathways including cAMP signaling, neuroactive ligand-receptor interaction, cytokine-cytokine receptor interaction, cell adhesion molecules, and calcium signaling pathway (Fig. 1b). Notably, cAMP signaling and calcium signaling pathway were reported to play a critical role in stem cell migration [15, 16].

### Cav3.2 T-type calcium channel was activated to enhance MSC-H cell migration

**Fig. 2:**
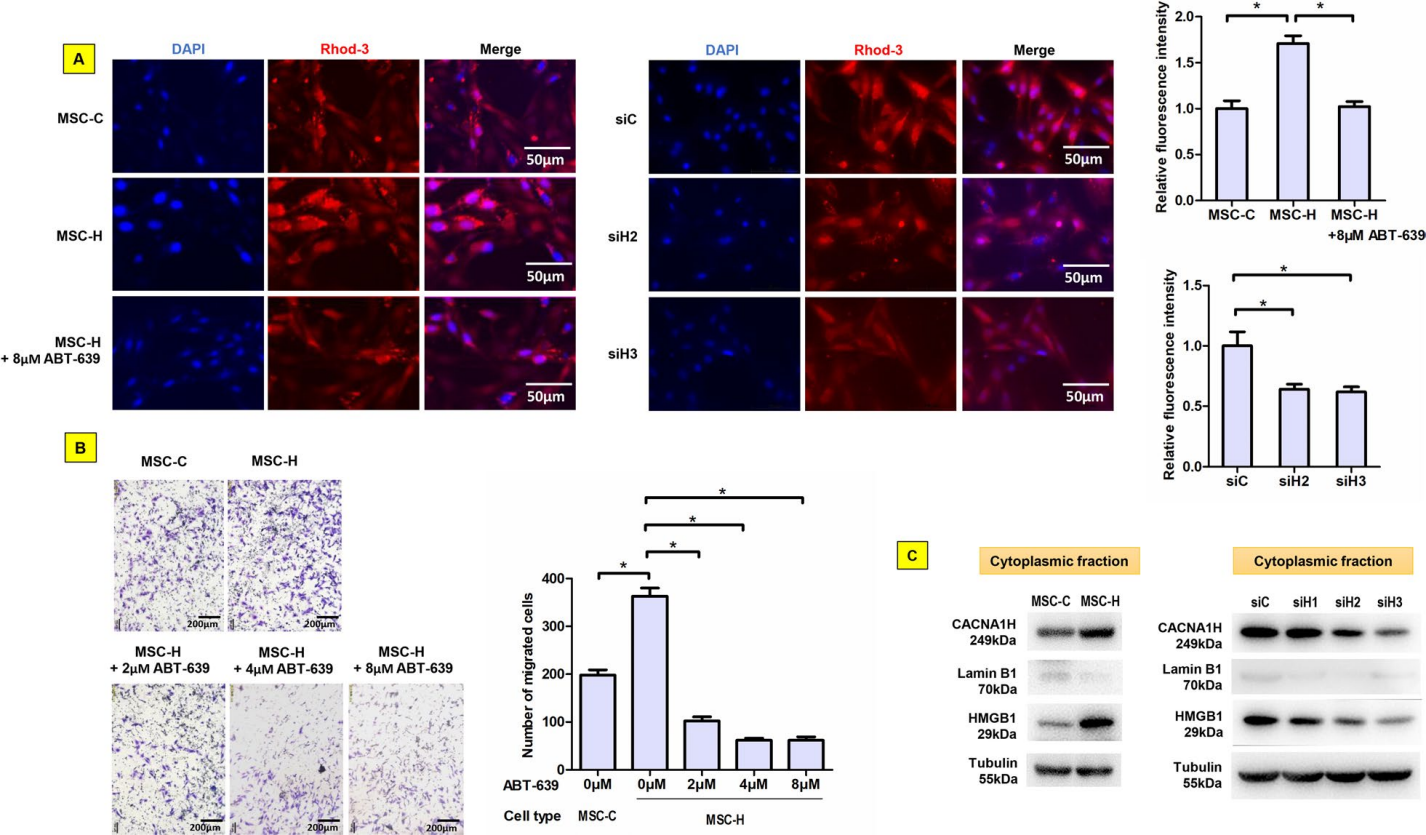
Contribution of Cav3.2 T-type calcium channel to MSC-H cell migration. Rhod-3 probes emitted red fluorescence when reacting with intercellular free calcium. MSC-H cells displayed higher fluorescent intensity than MSC-C cells, suggesting a significant increase of intercellular free calcium in MSC-H cells. But the fluorescence was substantially abrogated by either blockage of Cav3.2 T-type calcium channel with 8 μmol/l ABT-639 or HMGB1 knock-down as shown in siH2 and siH3 cells (**a**). In transwell assay, the population of migrated cells was increased in MSC-H cells when compared to MSC-C cells but diminished with increasing doses of ABT-639 (**b**). MSC-H cells showed a significant increase of cytoplasmic HMGB1. Knock-down of HMGB1 markedly reduced cytoplasmic HMGB1 level as shown in siH2 and siH3 cells. The siH1 cells had little change of cytoplasmic HMGB1 expression and therefore was excluded from further studies. The expression of Cav3.2 T-type calcium channel encoded by CACNA1H was increased 1.5-fold in MSC-H cells but inhibited by HMGB1 knock-down as shown in siH2 and siH3 cells (**c**)

Since microarray analysis revealed the association of calcium signaling pathway with HMGB1 upregulation, we investigated intracellular free calcium and calcium channel activity in MSC-H cells. In calcium imaging, the red fluorescence from Rhod-3 calcium probes was visible intracellularly and varied with significantly higher intensity in MSC-H cells than MSC-C cells (Fig. 2a). The increase of intracellular free calcium was correlated with the enhanced cell migration of MSC-H cells in transwell assay (Fig. 2b). Next, the activity of calcium channels was investigated to whether the calcium channel-mediated calcium influx was responsible for the elevation of intracellular free calcium. Three gene transcripts encoding calcium channels were identified by microarray to increase more than 1.5-fold in MSC-H cells. However, Western blot analysis demonstrated that Cav 3.2 T-type calcium channel encoded by CACNA1H gene was the only protein of stably overexpressed in MSC-H cells (Fig. 2c, Additional file 2: Fig. 2c) while there were no obvious changes for other transcripts. Intracellular free calcium was greatly reduced by treating MSC-H cells with ABT-639, a selective blocker of Cav 3.2 T-type calcium channel (Fig. 2a). Moreover, CACNA1H upregulation was suggested to contribute significantly to high mobility of MSC-H cells given that ABT-639 significantly inhibited MSC-H cell migration in a dose-dependent manner (Fig. 2b). In order to elucidate the correlation between HMGB1 and CACNA1H expression, we constructed three plasmids expressing shRNAs targeting HMGB1 and transfected them into MSC-H cells. Suppression of HMGB1 resulted in downregulation of CACNA1H (Fig. 2c) and decrease of intracellular free calcium in MSC-H cells (Fig. 2a).

### γ-cystathionase/H_2_S signaling was involved in the activation of Cav3.2 T-type calcium channel in MSC-H cells

**Fig. 3:**
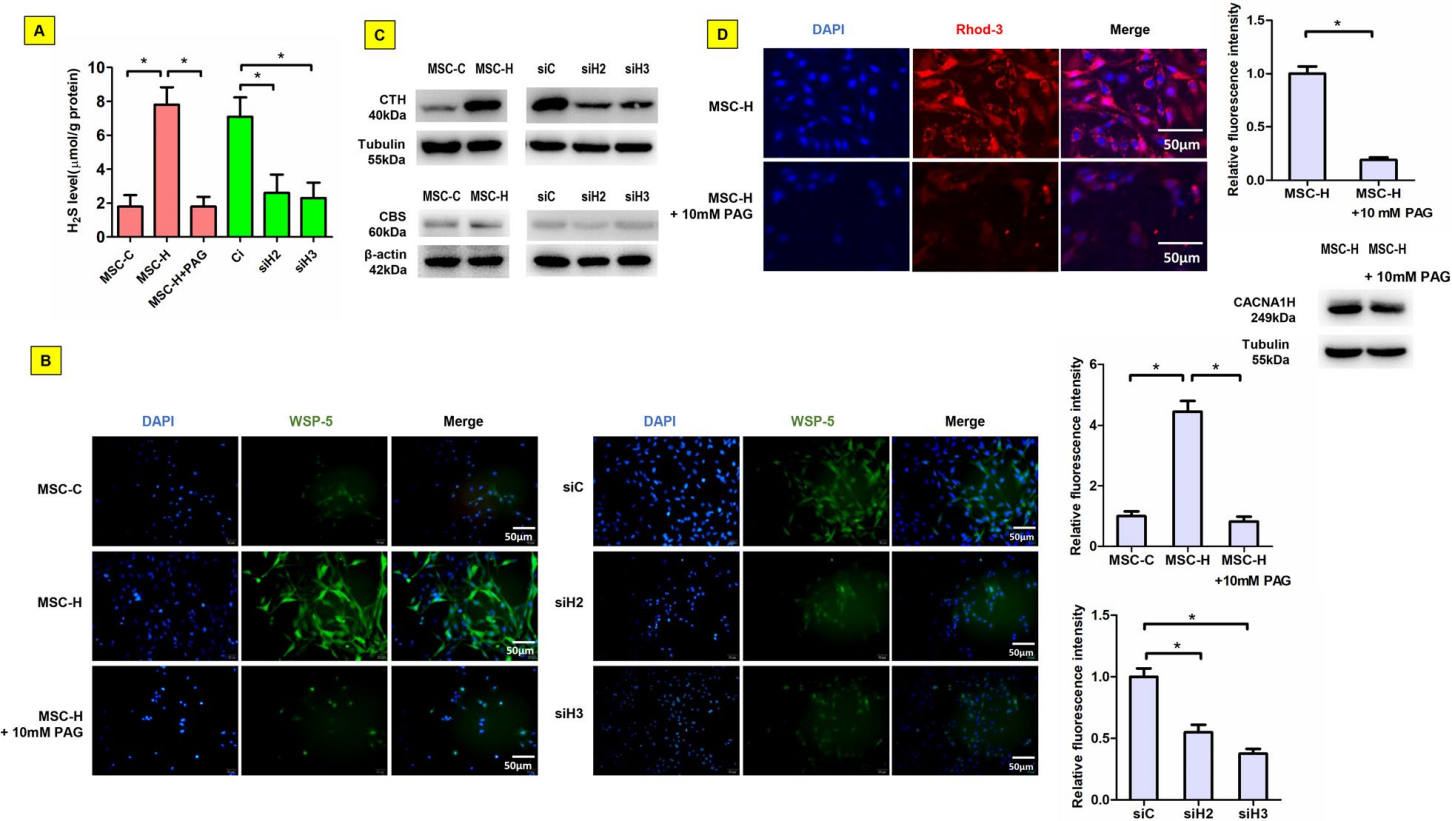
Activation of Cav3.2 T-type calcium channel by γ-cystathionase (CTH) /H_2_S signaling in MSC-H cells. MSC-H cells had fourfold higher H_2_S level in the culture supernatant than MSC-C cells. The extracellular H_2_S level was reduced in siH2 and siH3 cells with HMGB1 knock-down. When MSC-H cells were treated with 10 mmol/L PAG to inhibit CTH activity, H_2_S level in the culture supernatant was greatly decreased (**a**). Intracellular H_2_S level was assayed by using WSP-5 probes which reacted with intracellular H_2_S to emit green fluorescence. The fluorescent intensity was significantly higher in MSC-H cells than MSC-C cells, suggesting a remarkable increase of intracellular H_2_S in MSC-H cells. The intracellular H_2_S diminished when MSC-H cells was treated with 10 mmol/l PAG or subjected to HMGB1 knock-down as shown in siH2 and siH3 cells (**b**). The expression of CTH was increased in MSC-H cells but inhibited by HMGB1 knock-down as shown in siH2 and siH3 cells. Cystathionine-β-synthase (CBS) remained at a low level in all study groups regardless of HMGB1 overexpression and knock-down (**c**). Blockage of CTH by 10 mmol/l PAG reduced the fluorescent intensity of Rhod-3 probes without change of CACNA1H expression (**d**), suggesting a pivotal role of CTH on regulation of intracellular free calcium in MSC-H cells. The asterisk indicated a *P* value < 0.05

Although CACNA1H upregulation was suggested to account for the change of intracellular free calcium in MSC-H cells, the obvious increase of intracellular free calcium ion was not likely to be fully explained by nearly 1.5-fold increase of CACNA1H expression after HMGB1 upregulation. Therefore, we postulated that Cav3.2 T-type calcium channel was presumably activated via signaling pathways other than translational stimulation. After culture for 48 h, H_2_S level in supernatant was increased fourfold higher for MSC-H cells than MSC-C cells (Fig. 3a). The intracellular H_2_S was indicated by WSP-5 probes emitting green fluorescence after reacting with H_2_S. The intensity was significantly higher in MSC-H cells than MSC-C cells, suggesting the production and extracellular release of H_2_S after HMGB1 upregulation (Fig. 3b). Since biosynthesis of H_2_S in mammals largely depended upon catalysis of cystathionine-β-synthase (CBS) and CTH, the expression of the two enzymes was assayed to determine which one was responsible for the synthesis of H_2_S by MSC-H cells. Although both enzymes were expressed at a low level in MSC-C cells, the expression of CTH was exclusively increased in MSC-H cells (Fig. 3c, Additional file 2: Fig. 3c). Moreover, inhibition of CTH activity by DL-propargylglycine (PAG) reduced H_2_S synthesis (Fig. 3a, b). The intracellular level of free calcium was decreased in MSC-H cells after PAG treatment although the expression of CACNA1H was not altered (Fig. 3d). To justify the stimulatory effect of HMGB1 on CTH expression, the expression of CTH was assayed after knock-down of HMGB1 in MSC-H cells. The siH2 and siH3 cells had significantly lower levels of CTH than the siC cells (Fig. 3c), which was consistent with reduced H_2_S production after HMGB1 knock-down (Fig. 3a, b).

### The regulation of CTH activity by interaction with HMGB1 depended on redox state of HMGB1

**Fig. 4:**
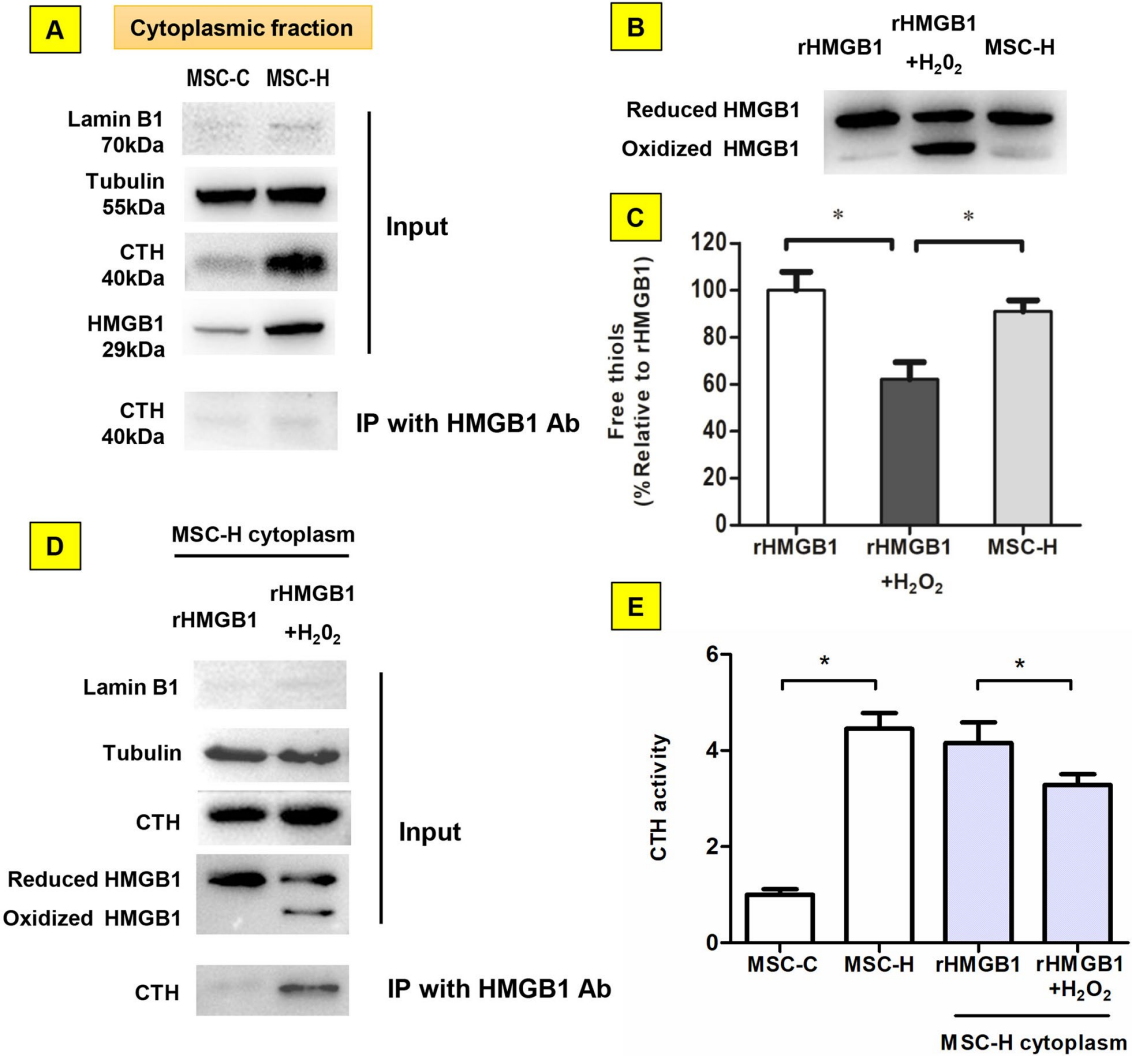
Regulation of γ-cystathionase (CTH) activity by HMGB1 upon oxidation. **a** Almost no CTH was co-immunoprecipitated by the bait protein HMGB1 from the cytoplasm of MSC-C and MSC-H cells. **b**, **c** The redox state of HMGB1 from the cytoplasm of MSC-H cells was analyzed by Western blot and protein free thiol assay. The partially oxidized HMGB1 sample with approximately 50% of HMGB1 oxidized (rHMGB1 + H_2_O_2_) was obtained by incubation the reduced HMGB1 standard (rHMGB1) with 50 μmol/l hydrogen peroxide for 1 h on ice. The immunoblotting by HMGB1 antibody yielded two bands of equal chemiluminescent intensity from the sample of rHMGB1 + H_2_O_2_ in comparison to a single band from rHMGB1. Intriguingly, the cytoplasmic extract from MSC-H cells was assayed to show a single band of HMGB1 corresponding to rHMGB1, suggesting cytoplasmic HMGB1 of MSC-H cells remained non-oxidized (B). HMGB1 was isolated from the cytoplasm of MSC-H cells by immunoprecipitation with anti-flag antibody. The purified HMGB1 contained high amount of free thiol, resembling rHMGB1 and significantly higher than the free thiol content of rHMGB1 + H_2_O_2_ (**c**). The MSC-H cytoplasm was mixed with rHMGB1 + H_2_O_2_ and rHMGB1, respectively, at 20:1 ratio of total protein. The HMGB1-associated protein was co-immunoprecipitated with HMGB1 antibody and analyzed by immunoblotting with anti-CTH antibody. A significant amount of CTH was co-immunoprecipitated from the mixture of MSC-H and rHMGB1 + H_2_O_2_. In comparison, co-immunoprecipitation yielded little CTH from the mixture of MSC-H and rHMGB1 (**d**). The CTH activity was determined by the efficiency of catalyzing α,γ-elimination reaction of L-cystathionine to produce α-ketobutyrate. The cytoplasm of MSC-H cells had a higher level of CTH activity than MSC-C cells. Adding rHMGB1 + H_2_O_2_ to MSC-H cytoplasm reduced the CTH activity, but rHMGB1 had no such effect (**e**). The asterisk indicated a *P* value < 0.05

The interaction of CTH and HMGB1 was assayed to determine whether this was responsible for the activation of CTH in MSC-H cells. The co-immunoprecipitation plus immunoblotting assay yielded trace CTH immunoprecipitated by HMGB1 from the cytoplasm of MSC-C and MSC-H cells (Fig. 4a, Additional file 2: Fig. 4a). Intriguingly, the interaction of CTH with HMGB1 was demonstrated previously in liver ischemic reperfusion injury which was pathologically driven by oxidative stress [12]. Notably, bioactive HMGB1 had two redox isoforms depending on redox status of environment where it existed. Immunoblotting of cytoplasmic protein from MSC-H cells showed a single band corresponding to the rHMGB1 (Fig. 4b, Additional file 2: Fig. 4b). The free thiol content of cytoplasmic HMGB1 from MSC-H cells was comparable to the rHMGB1 standard (Fig. 4c, Additional file 2: Fig. 4c), verifying that HMGB1 overexpressed in MSC-H cells remained non-oxidized. Therefore, we postulated that HMGB1 upon oxidation bound to CTH. The sample of approximately 50% oxidized HMGB1 (rHMGB1 + H_2_O_2_) was prepared from incubating the rHMGB1 standard with 50 μmol/L hydrogen peroxide [13]. The MSC-H cytoplasm was mixed either with rHMGB1 or with rHMGB1 + H_2_O_2_ at 20:1 ratio of total protein and sent to co-immunoprecipitation with HMGB1 antibody. The immunoblotting analysis confirmed that a significant amount of CTH was co-precipitated with HMGB1 from the mixture of rHMGB1 + H_2_O_2_ and MSC-H cytoplasm. In comparison, the co-immunoprecipitation yielded little CTH from the mixture of rHMGB1 and MSC-H cytoplasm (Fig. 4d). Moreover, the in vitro assay demonstrated that cytoplasmic CTH activity was increased in MSC-H cells but inhibited by adding rHMGB1 + H_2_O_2_ (Fig. 4e). Our findings suggested that the CTH activity was regulated by interaction with HMGB1 depending on HMGB1 redox state.

### β-Catenin was activated to increase CTH promoter activity in MSC-H cells

**Fig. 5:**
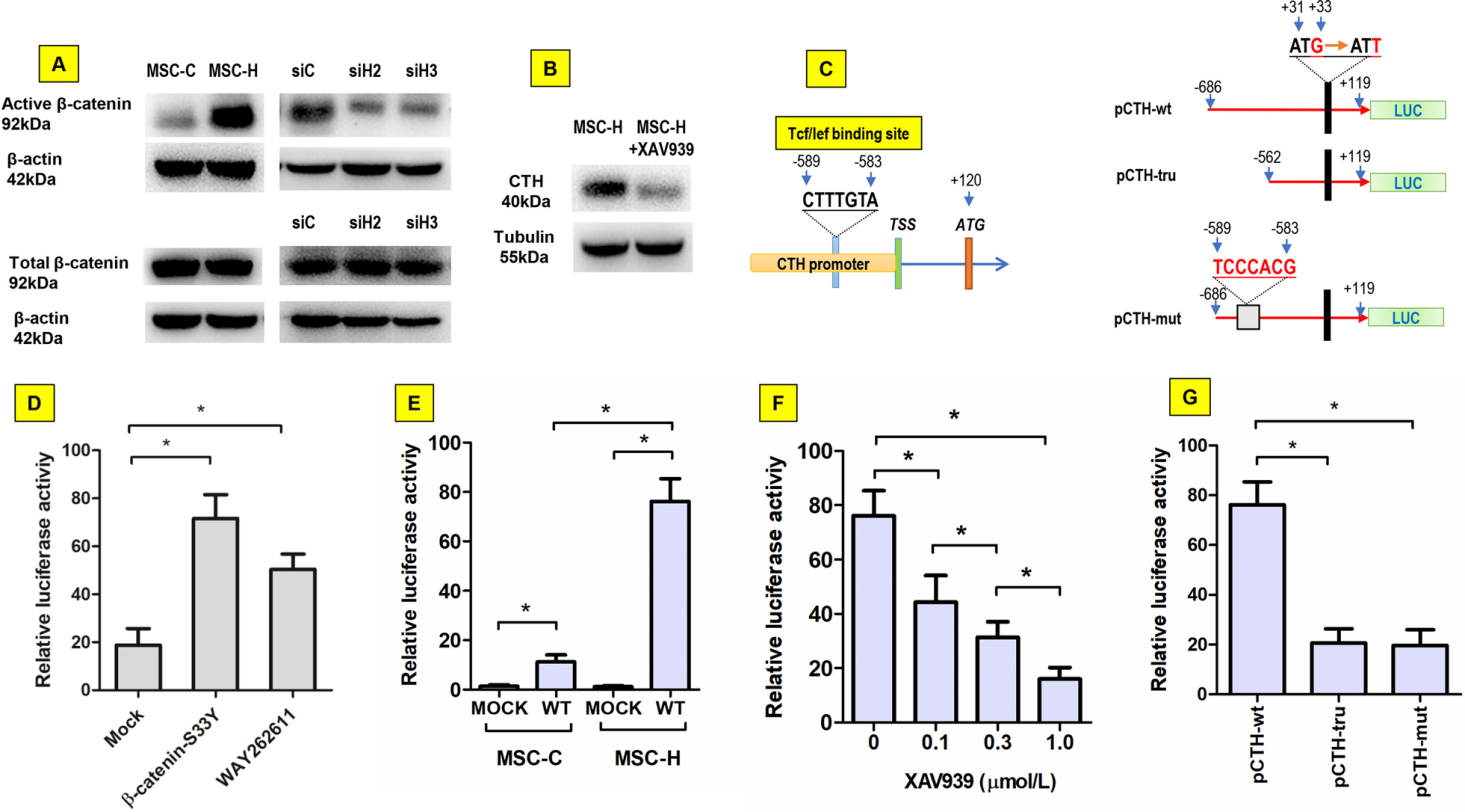
Regulation of γ-cystathionase (CTH) transcriptional activity by Wnt/β-catenin signaling. The active isoform of β-catenin was increased in MSC-H cells although total β-catenin level was not changed. HMGB1 knock-down reduced active β-catenin without effect on total β-catenin expression as shown in siH2 and siH3 cells (**a**). Inhibition of Wnt/catenin signaling by 1.0 μmol/l XAV939 suppressed the CTH expression in MSC-H cells (**b**). A conserved TCF/LEF binding sequence 589 bp upstream of transcriptional start site was identified on CTH promoter by bioinformatics analysis. The wild-type CTH promoter was cloned into luciferase reporter plasmid (pCTH-wt). To validate the effect TCF/LEF binding element on promoter activity, the plasmids of pCTH-mut and pCTH-tru were constructed by introducing site-specific mutation and upstream truncation of the putative TCF/LEF binding site into CTH promoter, respectively (**c**). In dual luciferase assay, the relative luciferase activity of pCTH-wt was significantly increased by co-transfection of pCMV-S33Y-β-catenin into 293 T cells when compared to the mock plasmid. Moreover, stimulation of 293 T cells with 1.0 μmol/l WAY262611 also enhanced the luciferase activity of pCTH-wt (**d**). The luciferase activity of pCTH-wt was significantly higher in MSC-H cells than MSC-C cells (**e**). XAV939 reduced the luciferase activity of pCTH-wt in MSC-H cells in a dose-dependent manner (**f**). The pCTH-wt plasmid had significantly higher luciferase activity in MSC-H cells than pCTH-mut and pCTH-tru (**g**). The asterisk indicated a *P* value < 0.05

β-catenin was assayed to determine whether CTH was activated by Wnt/β-catenin signaling. The active β-catenin was greatly increased in MSC-H cells although there was not obvious change of total β-catenin. HMGB1 knock-down inhibited the active isoform of β-catenin but had no effect on total β-catenin (Fig. 5a, Additional file 2: Fig. 5a). When Wnt/β-catenin signaling was blocked by 1.0 μmol/l XAV939, the expression of CTH was inhibited significantly (Fig. 5b, Additional file 2: Fig. 5b). Next, the activity of CTH promoter was determined by luciferase reporter assay given that a TCF/LEF binding site 589 bp upstream of transcriptional start site was identified by bioinformatics analysis (Fig. 5c). When the pCTH-wt plasmid was co-transfected with pCMV-S33Y-β-catenin into 293 T cells, the promoter activity was obviously increased. Similarly, stimulation of 293 T cells with 1.0 μmol/l Wnt/β-catenin agonist WAY262611 enhanced the promoter activity of pCTH-wt, suggesting that CTH promoter had a positive response to β-catenin activation (Fig. 5d). Then pCTH-wt was transfected into either MSC-H cells or MSC-C cells. The promoter activity was expectedly increased in the MSC-H cells where active β-catenin was accumulated (Fig. 5e). Furthermore, when MSC-H cells were treated with Wnt/β-catenin inhibitor XAV939, the promoter activity of pCTH-wt diminished in a dose-dependent manner (Fig. 5f). Finally, the site-specific mutation or upstream truncation of the putative Tcf/Lef binding site markedly abrogated the β-catenin induced CTH promoter activation in MSC-H cells (Fig. 5g). Taken together, our findings suggested the CTH promoter activity was positively regulated by Wnt/β-catenin signaling in MSC-H cells.

### β-catenin was activated by cAMP/PKA signaling in MSC-H cells

**Fig. 6:**
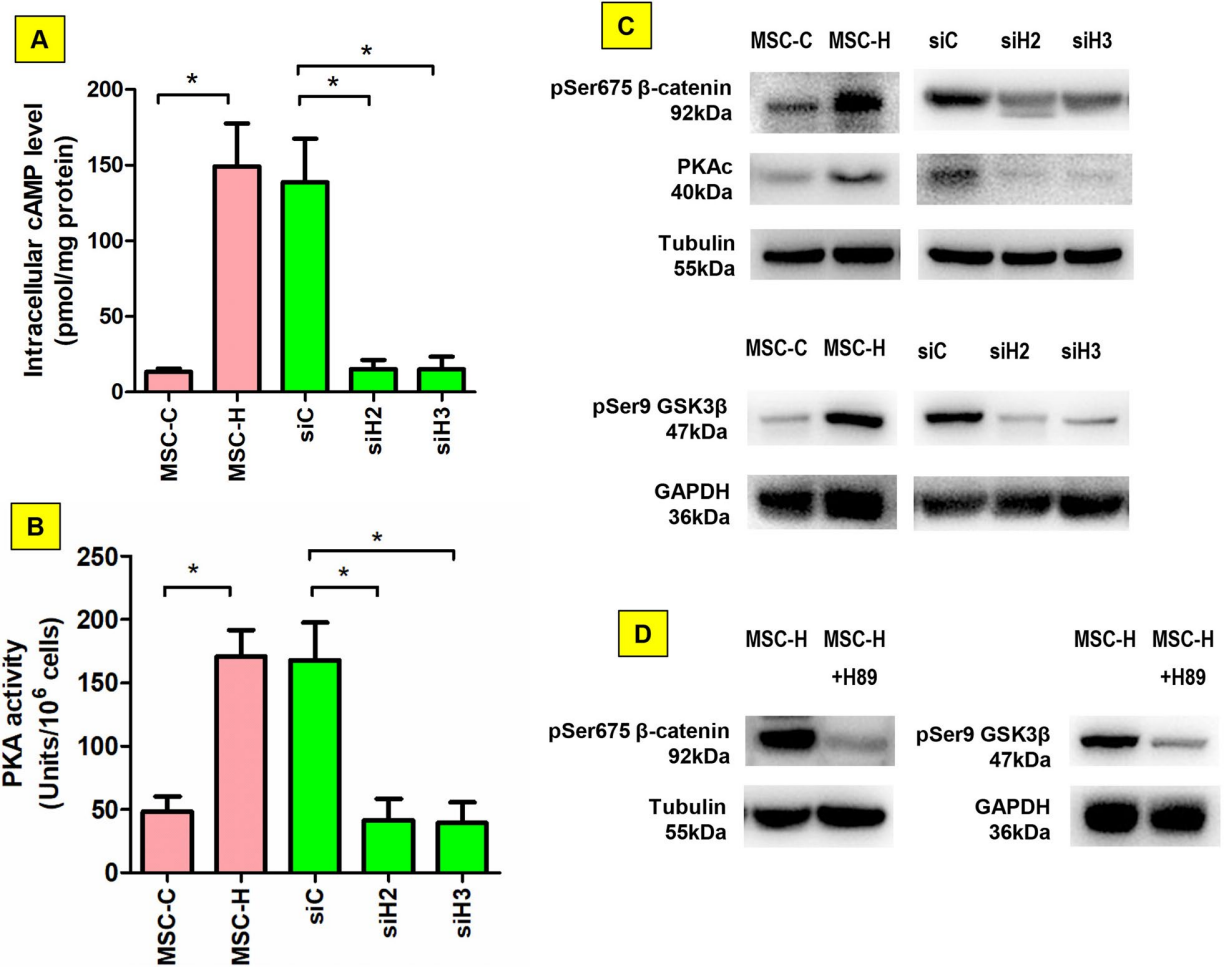
β-catenin activation by cAMP/PPKA signaling in MSC-H cells. Intracellular cAMP level was significantly higher in MSC-H cells than MSC-C cells. HMGB1 knock-down reduced intracellular cAMP as shown in siH2 and siH3 cells (**a**). In accordance, PKA activity was increased in MSC-H cells but inhibited by HMGB1 knock-down (**b**). The Ser675 of β-catenin and the Ser9 of GSK3β were increasingly phosphorylated, which was associated with high expression of PKA catalytic subunit in MSC-H cells. HMGB1 knock-down inhibited the expression of PKA catalytic subunit and phosphorylation of β-catenin and GSK3β. **c** Blockage of PKA by 10 μmol/l H89 inhibited phosphorylation of β-catenin at Ser657 and GSK3β at Ser9 in MSC-H cells (**d**). The asterisk indicated a *P* value < 0.05

Microarray analysis revealed that cAMP signaling was the key pathway modulated by HMGB1 in MSC-H cells (Fig. 1). The cAMP-dependent PKA was ubiquitous in mammalian cells and mediated biological processes by phosphorylation and activation of target proteins. Therefore, we assumed that β-catenin was activated by cAMP/PKA signaling in MSC-H cells. Firstly, intracellular cAMP level was assayed, demonstrating a significant increase in MSC-H cells when compared to MSC-C cells (Fig. 6a). HMGB1 knock-down remarkably decreased intracellular cAMP level. Similarly, the catalytic activity of PKA was promoted in MSC-H cells but abrogated by HMGB1 knock-down (Fig. 6b). In immunoblotting assay, PKA catalytic subunit was highly expressed in MSC-H cells, being positively correlated with high expression of pSer675 β-catenin which was intracellularly stable and transcriptionally active. Moreover, phosphorylation of glycogen synthase kinase 3β (GSK3β) was increased in MSC-H cells. HMGB1 knock-down inhibited the expression of PKA catalytic subunit, pSer675 β-catenin, and phosphorylated GSK3β (Fig. 6c, Additional file 2: Fig. 6c). Finally, upregulation of pSer675 β-catenin and phosphorylated GSK3β in MSC-H cells was inhibited by PKA inhibitor H89 (Fig. 6d, Additional file 2: Fig. 6d). Altogether, the cAMP/PKA signaling was activated and responsible for β-catenin activation in MSC-H cells.

## Discussion

The MSCs inherently migrate to chemotactic signals from injured tissue, which is as important as their capacity for self-renewal and differentiation, enabling them to mediate tissue repair and regeneration. Although the cell migration has been revealed to depend on the expression of chemotaxis factors like CXCR4 and CXCR7, the regulation of migratory response is ultimately governed by transmission of intercellular signals. Calcium is a ubiquitous intracellular messenger in many eukaryotic signal transduction cascades and has specific roles in regulation of cell excitability [17], exocytosis [18], motility [19], and apoptosis [20]. Stem cell migration is one of the key physiological processes regulated by intracellular calcium signal. The calcium signal induced by extracellular ATP stimulated MSC migration [16], and extremely low frequency electromagnetic fields promoted MSC migration by increasing intracellular calcium [21]. The actin-myosin cytoskeleton was regarded as the key regulatory target of intracellular calcium [22–24]. Non-muscle myosin II promoted MSC migration by inhibiting synthesis of extracellular matrix and adhesion molecules and driving cytoskeletal polarization [25, 26]. Reduction of free calcium in the cytosol and endoplasmic reticulum by silencing mitochondria calcium uniporter compromised cell migration by stiffening actin cytoskeleton, losing cell polarization, and impairing the dynamics of focal adhesion proteins [27]. During chemotactic migration, actin depolymerization inhibited protrusions of MSCs, highlighting the importance of actin assembly on efficient homing to target tissue [28]. Besides, intracellular calcium was discovered to steer cell migration by forming high-calcium microdomains, namely calcium flicker, in response to membrane tension and chemoattractant signal transduction [29]. The intermediate/big-conductance potassium channels mediated by intercellular calcium were suggested to orchestrate MSC migration as well as self-renewal and differentiation [21]. In our study, microarray analysis revealed that calcium signaling was activated in MSC-H cells (Fig. 1b), and the follow-up cell experiment confirmed that MSC-H cells had a higher intracellular level of free calcium than MSC-C cells (Fig. 2a). In accordance, the cell migration was increased in MSC-H cells (Fig. 2b). Intracellular calcium homeostasis is maintained through the functional interplay of calcium transport channel, calcium buffers, and sensors, and calcium transport channels play a pivotal role in calcium dynamics and signal transduction [30]. Cav3.2 T-type calcium channel was identified to upregulate in MSC-H cells. Cav3.2 T-type calcium channel is a T-type member of the α1 subunit family and a protein in the voltage-dependent calcium channel complex. The α-1 subunit has 24 transmembrane segments forming the pores for calcium influx and is the core structure of T-type calcium channels. Aberrant activation of Cav3.2 T-type calcium channel was associated with epilepsy [31], neuropathic pain [32], and tumor progression [33]. Here, we showed that blockage of Cav3.2 T-type calcium channel by ABT-639 abrogated intracellular calcium increases and cell migration in MSC-H cells. Moreover, HMGB1 knock-down significantly decreased intracellular level of free calcium and Cav3.2 T-type calcium channel, suggesting HMGB1 stimulated Cav3.2 T-type calcium channel to induce calcium influx and MSC-H cell migration.

However, the obvious changes of intracellular calcium could not be fully explained by 1.5-fold increase of CACNA1H expression in MSC-H cells. Therefore, we proposed that the activity of Cav3.2 T-type calcium channel would be increased in MSC-H cells by endogenous modulators such as H_2_S. The gas has a myriad of biological signaling functions and is produced in small amounts from cysteine by the enzymes CBS and CTH in eukaryotic cells. During cyclophosphamide-induced nociceptor excitation in urothelial cells, CTH/H_2_S signaling was activated to stimulate Cav3.2 T-type calcium channel by HMGB1 which was released from macrophages and bound to receptor for advanced glycation end-products [32]. Our investigation revealed that CTH but not CBS was remarkably increased in MSC-H cells, which was correlated with a significant increase of extracellular and intracellular H_2_S level (Fig. 3a, b). Moreover, inhibition of CTH by specific antagonist PAG blocked H_2_S production and intracellular calcium increase in MSC-H cells (Fig. 3a, b, d). HMGB1 knock-down suppressed CTH expression and subsequent H_2_S synthesis in MSC-H cells (Fig. 3a–c). Our findings supported that HMGB1 stimulated Cav3.2 T-type calcium channel-mediated calcium influx via CTH/H_2_S signaling.

A recent study identified CTH as HMGB1-associated protein during the hepatocellular response to ischemia reperfusion injury [12]. Thus, we assumed that HMGB1 might modulate CTH activity via binding to CTH. However, the co-immunoprecipitation plus immunoblotting yielded no obvious CTH being immunoprecipitated with HMGB1 antibody from MSC-H cell lysates (Fig. 4a). Since the interaction of CTH and HMGB1 was previously discovered under ischemia reperfusion injury pathologically characterized by strong oxidative stress, our findings opposing to the existing literature were presumably due to thiol redox transition of HMGB1 under different redox conditions. Therefore, redox state of HMGB1 was analyzed in MSC-H cells, and the standard with and without hydrogen peroxide pretreatment served as oxidized and non-oxidized HMGB1 control, respectively. In the immunoblotting, cytoplasmic protein from MSC-H cells showed a single band corresponding to rHMGB1 (Fig. 4b). Furthermore, the free thiol content of HMGB1 in MSC-H cells was equivalent to rHMGB1 (Fig. 4c). After adding rHMGB1 + H_2_O_2_ to the MSC-H cytoplasmic extract, a significant amount of CTH was precipitated with HMGB1 antibody which was correlated with inhibition of CTH activity (Fig. 4d, e). Thus, CTH specifically interacted with oxidized HMGB1, and HMGB1 produced from MSC-H cells remained non-oxidized rendering no reaction with CTH.

To further elucidate the mechanisms that underlay the activation of CTH in MSC-H cells, CTH promoter was analyzed by bioinformatics to search putative binding site of transcriptional factors. A conserved TCF/LEF binding site was identified in CTH promoter and located at 583–589 bp upstream of transcription start site (Fig. 5c). The luciferase reporter assay confirmed that CTH promoter had positive response to β-catenin (Fig. 5d). In canonical Wnt/β-catenin signaling, plenty of active β-catenin proteins translocate into the nucleus and bind to members of the TCF/LEF family of transcription factors to mediate the transcriptional response. Therefore, Wnt/β-catenin signaling was postulated to regulate CTH transcriptional activity in MSC-H cells. Although the total amount of β-catenin was not changed, a substantial increase of active β-catenin was discovered and correlated with the enhanced activity of CTH promoter in MSC-H cells (Fig. 5a, e). HMGB1 knock-down reduced active β-catenin in MSC-H cells (Fig. 5a). Moreover, blockage of Wnt/β-catenin signaling by XAV939 significantly suppressed CTH promoter activity and protein expression in MSC-H cells (Fig. 5b, f), suggesting that Wnt/β-catenin signaling mediated HMGB1-induced CTH transcriptional activation. The follow-up luciferase reporter assay showed that the integrity of TCF/LEF binding site was needed for CTH promoter activity. Site-specific mutation of the TCF/LEF binding site abrogated the activity of CTH promoter (Fig. 5g). The TCF/LEF binding site was located at the upstream of key regulatory elements of CTH promoter. Therefore, upstream truncation of the TCF/LEF binding site yielded the similar result as the site mutation (Fig. 5g).

Finally, the association of PKA and Wnt/β-catenin signaling was investigated, given that microarray revealed that cAMP signaling was the most enriched pathways in MSC-H cells. PKA is the main intracellular target for cAMP, and when sensing high cAMP levels, converts from the inactive tetrameric holoenzyme to the active dissociated catalytic subunit. The follow-up cell experiment revealed that intracellular cAMP level was increased along with the upregulation of PKA catalytic activity in MSC-H cells. HMGB1 knock-down reduced the cAMP level as well as PKA activity (Fig. 6a, b). Active PKA regulates the downstream signaling cascades by phosphorylation of specific serine and threonine residues on target proteins. Wnt/β-catenin signaling was reported to be upregulated by PKA through two pathways: phosphorylation of GSK3β [34] and β-catenin at Ser675 [35]. GSK3β is the crucial component of β-catenin destruction complex in the cytoplasm, targeting β-catenin for ubiquitin-mediated degradation. Phosphorylation of GSK3β dissociates the β-catenin destruction complex, resulting in stabilization of cytoplasmic β-catenin to promote Wnt/β-catenin-mediated transcriptional activity. Moreover, the stability of β-catenin is substantially improved by phosphorylation of β-catenin at Ser675. The two distinct pathways are not mutually exclusive but often work together to promote Wnt/β-catenin signaling [36]. Our study revealed that both phosphorylated GSK3β and phospho-Ser675 β-catenin were increased in MSC-H cells, which was suppressed by PKA specific inhibitor H89 (Figs. 5a, 6c, d). Therefore, Wnt/β-catenin signaling was activated by cAMP/PKA in MSC-H cells.

It might be argued that cAMP/PKA stimulated Cav3.2 T-type calcium channel by phosphorylation of Ser1107 in the II-III loop of the channel protein [37]. This was not contrary to our findings but provided further evidence for PKA-dependent activation of downstream Cav3.2 T-type calcium channel. Limitation of the study included lack of evidence from in vivo experiments, and the reason why HMGB1 triggered cAMP signaling need additional investigation.

## Conclusion

**Fig. 7:**
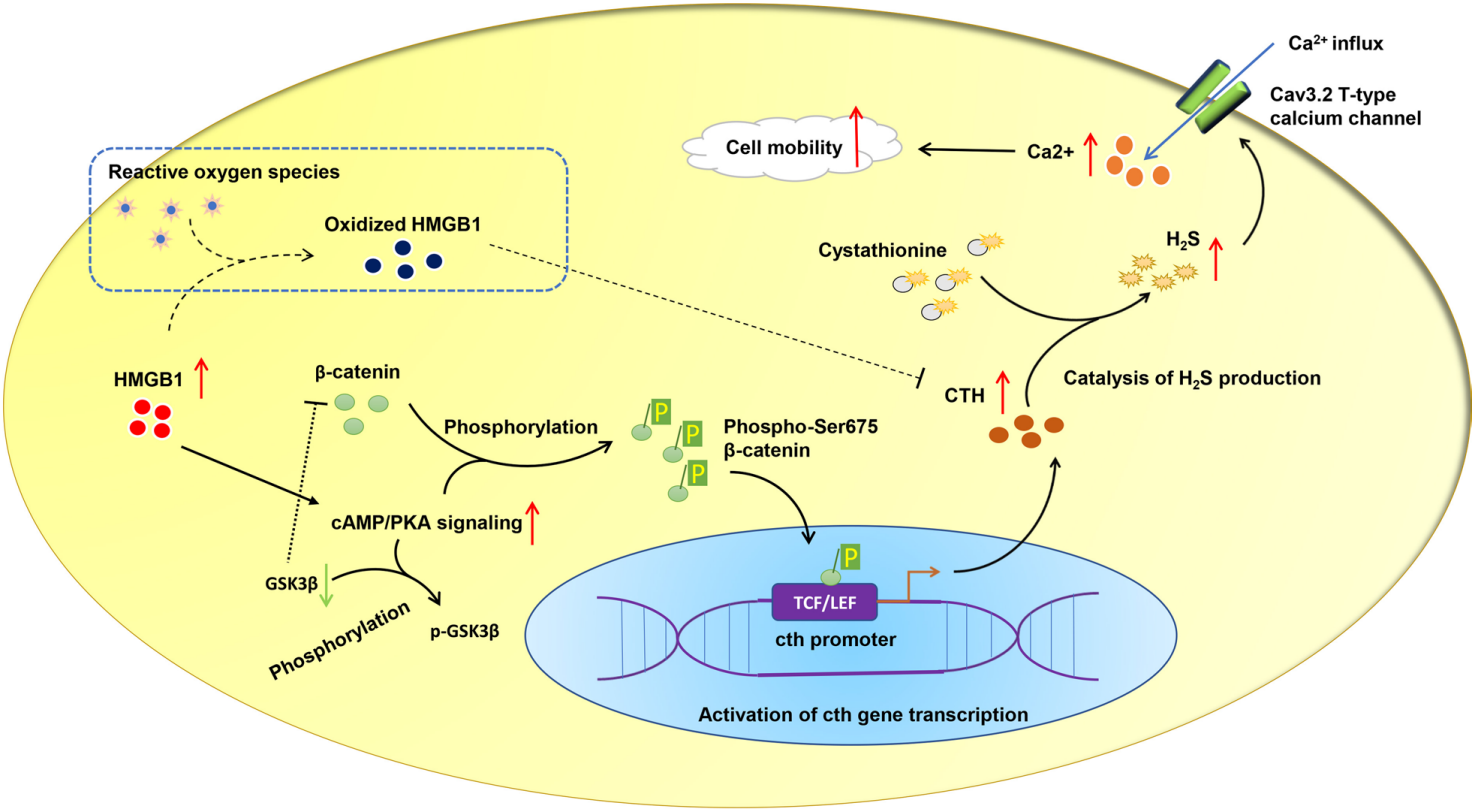
Illustration of mechanisms that HMGB1 modification improves the mobility of mesenchymal stem cells. HMGB1 activates cAMP/PKA to stabilize β-catenin by phosphorylation of GSK3β and β-catenin at Ser675, enabling β-catenin to accumulate intracellularly and translocate into the nucleus. The CTH promoter is thereafter activated by interaction of β-catenin with TCF/LEF, stimulating gene transcription. Cytoplasmic CTH catalyzes endogenous production of hydrogen sulfide from organic sulfide substrates like cystathionine. The Cav 3.2 T-type calcium channel is activated by hydrogen sulfide to drive extracellular calcium influx, and calcium signaling pathway is subsequently activated to promote cell mobility. Besides, HMGB1 presumably interacts with CTH to inhibit the catalysis of hydrogen sulfide production when it is oxidized by oxidative stress

Our study revealed that modification of MSCs with HMGB1 promoted cAMP/PKA mediated β-catenin stabilization and nuclear translocation, which in turn increased the transcription of γ-cystathionase, subsequent H_2_S synthesis and calcium influx mediated by Cav3.2 T-type calcium channel (Fig. 7). The regulatory importance of this signaling pathway was highlighted in the cellular mobility of MSC-H cell line.

## Supplementary Information

**Additional file 1:** Microarray data.

**Additional file 2:** Western blot images.

## Data Availability

The datasets generated and/or analyzed during the current study are available upon request to the corresponding authors.

## Abbreviations

β-catenin-S33Y: β-Catenin mutant with Ser 33 replaced by Tyr 33
cAMP: Cyclic adenosine monophosphate
CBS: Cystathionine β synthase
CTH: γ-Cystathionase, cystathionine γ lyase
DAPI: 4′,6-Diamidino-2-phenylindole
DMEM: Dulbecco’s modified Eagle’s medium
GSK3β: Glycogen synthase kinase 3β
GO: Gene ontology
HMGB1: High mobility group box 1
HMGB1 Ab: HMGB1 antibody
IP: Immunoprecipitation
H_2_S: Hydrogen sulfide
H_2_O_2_: Hydrogen peroxide
KEGG: Kyoto Encyclopedia of Genes and Genomes
MSC: Mesenchymal stem cell
MSC-C: Mesenchymal stem cells transfected with empty vector
MSC-H: HMGB1 modified mesenchymal stem cell
PAG: DL-propargylglycine
PKA: Protein kinase A
pCTH-wt: Luciferase reporter vector carrying wild-type CTH promoter
pCTH-mut: Luciferase reporter vector carrying CTH promoter with mutated TCF/LEF binding site
μM: μmol/l
mM: mmol/l
pCTH-tru: Luciferase reporter vector carrying truncated CTH promoter
pSer675 β catenin: Ser 675-phosphorylated β catenin, β catenin phosphorylated at Ser 675
rHMGB1: Reduced HMGB1
siC: Vehicle control plasmid
siH: Plasmid carrying shRNA targeting HMGB1
