# Supplementary material for: Modification of mesenchymal stem cells by HMGB1 promotes the activity of Cav3.2 T-type calcium channel via PKA/β-catenin/γ-cystathionase pathway
Source: Stem Cell Res Ther. 2022 Jan 10;13:4. doi: 10.1186/s13287-021-02677-z (PMC8744322; doi:10.1186/s13287-021-02677-z)
Supplement: Supplementary file 2 — Additional file 2: Western blot images. [file 13287_2021_2677_MOESM2_ESM.pptx]

## Slide 1
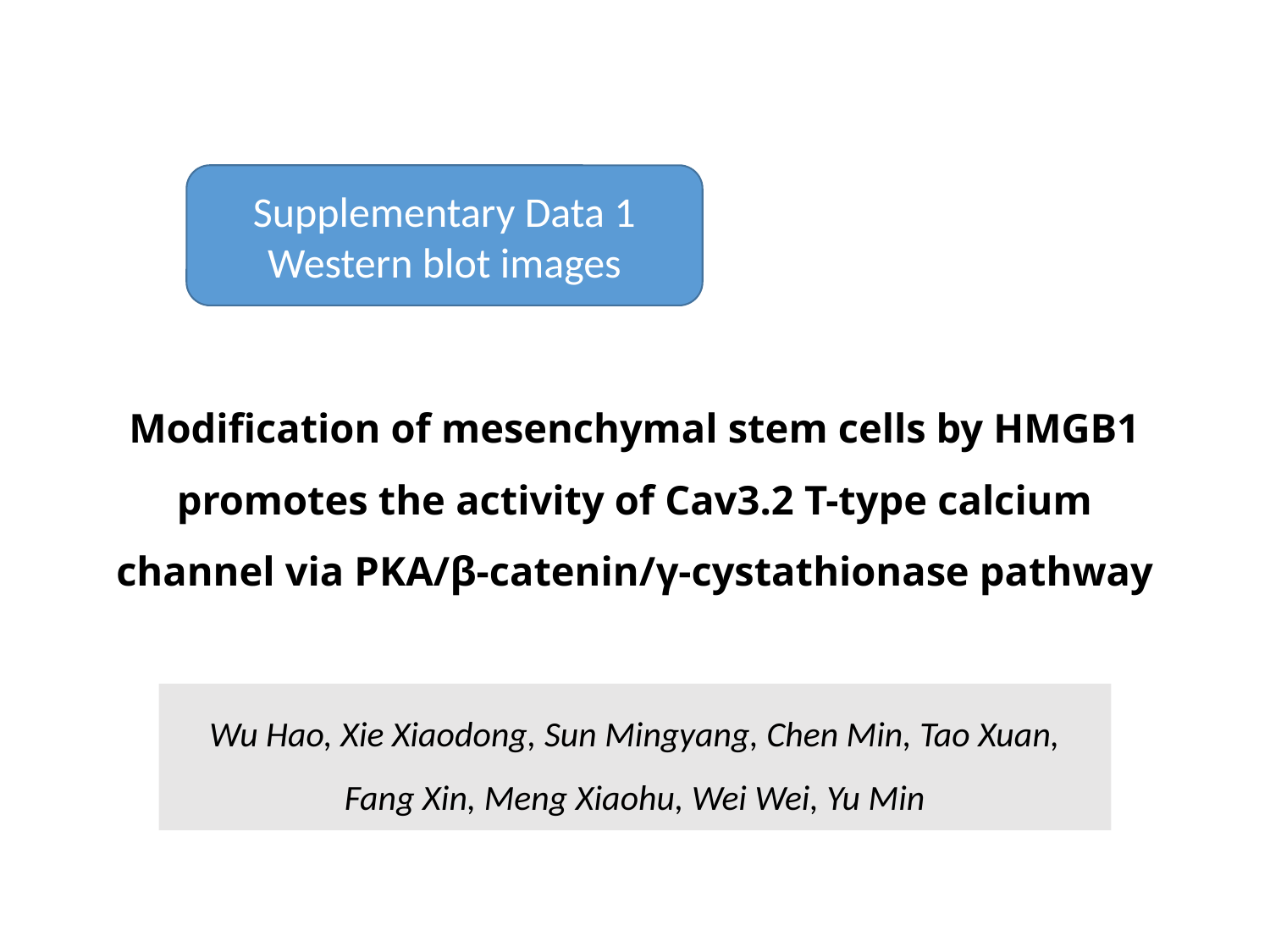

Supplementary Data 1
Western blot images
# Modification of mesenchymal stem cells by HMGB1 promotes the activity of Cav3.2 T-type calcium channel via PKA/β-catenin/γ-cystathionase pathway
Wu Hao, Xie Xiaodong, Sun Mingyang, Chen Min, Tao Xuan, Fang Xin, Meng Xiaohu, Wei Wei, Yu Min

## Slide 2
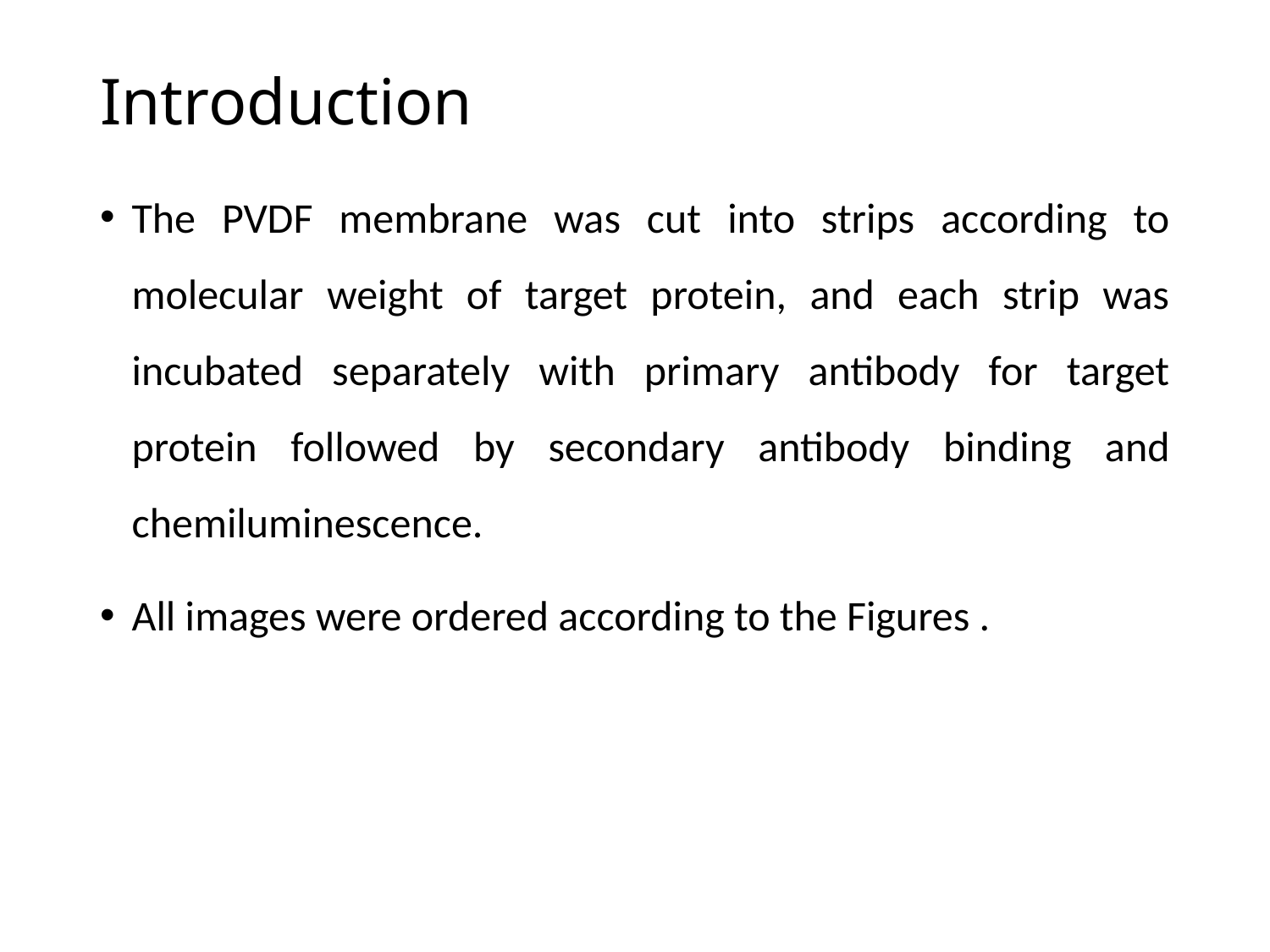

# Introduction
The PVDF membrane was cut into strips according to molecular weight of target protein, and each strip was incubated separately with primary antibody for target protein followed by secondary antibody binding and chemiluminescence.
All images were ordered according to the Figures .

## Slide 3
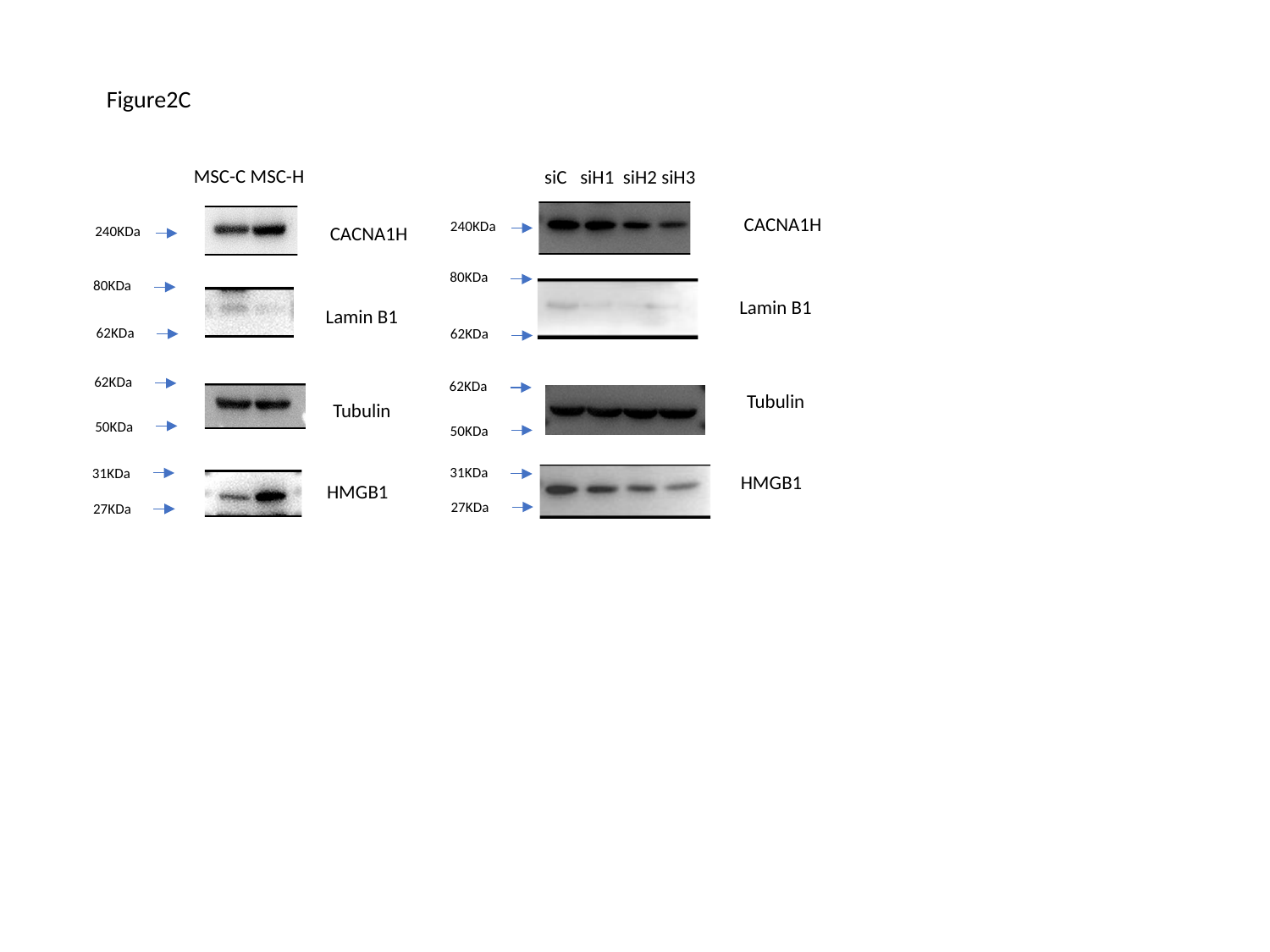

Figure2C
MSC-C MSC-H
siC siH1 siH2 siH3
CACNA1H
240KDa
CACNA1H
240KDa
80KDa
80KDa
 Lamin B1
 Lamin B1
62KDa
62KDa
62KDa
62KDa
Tubulin
Tubulin
50KDa
50KDa
31KDa
31KDa
HMGB1
HMGB1
27KDa
27KDa

## Slide 4
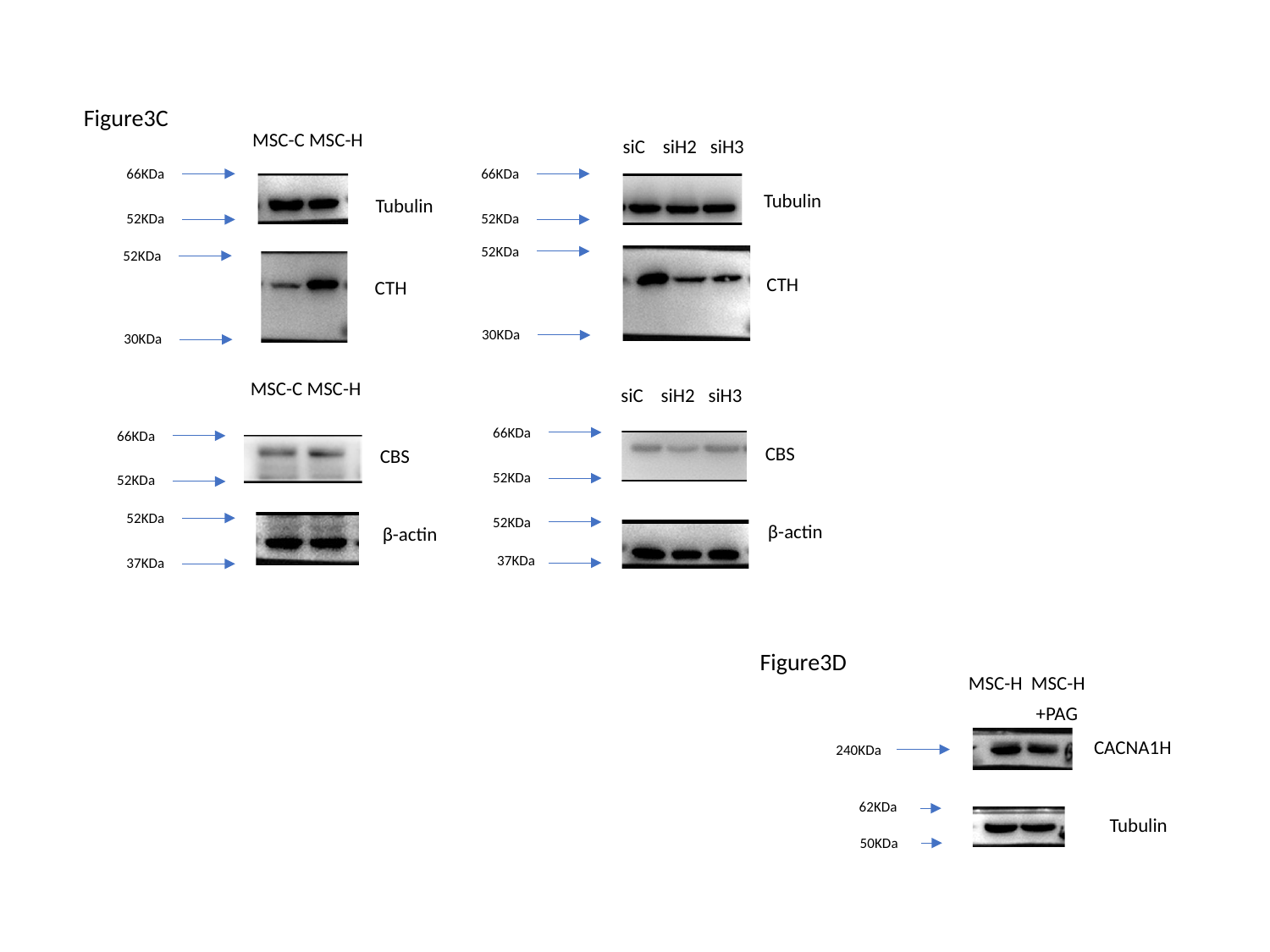

Figure3C
MSC-C MSC-H
siC siH2 siH3
66KDa
66KDa
Tubulin
Tubulin
52KDa
52KDa
52KDa
52KDa
CTH
CTH
30KDa
30KDa
MSC-C MSC-H
siC siH2 siH3
66KDa
66KDa
CBS
CBS
52KDa
52KDa
52KDa
52KDa
 β-actin
 β-actin
37KDa
37KDa
Figure3D
MSC-H MSC-H
+PAG
CACNA1H
240KDa
62KDa
Tubulin
50KDa

## Slide 5
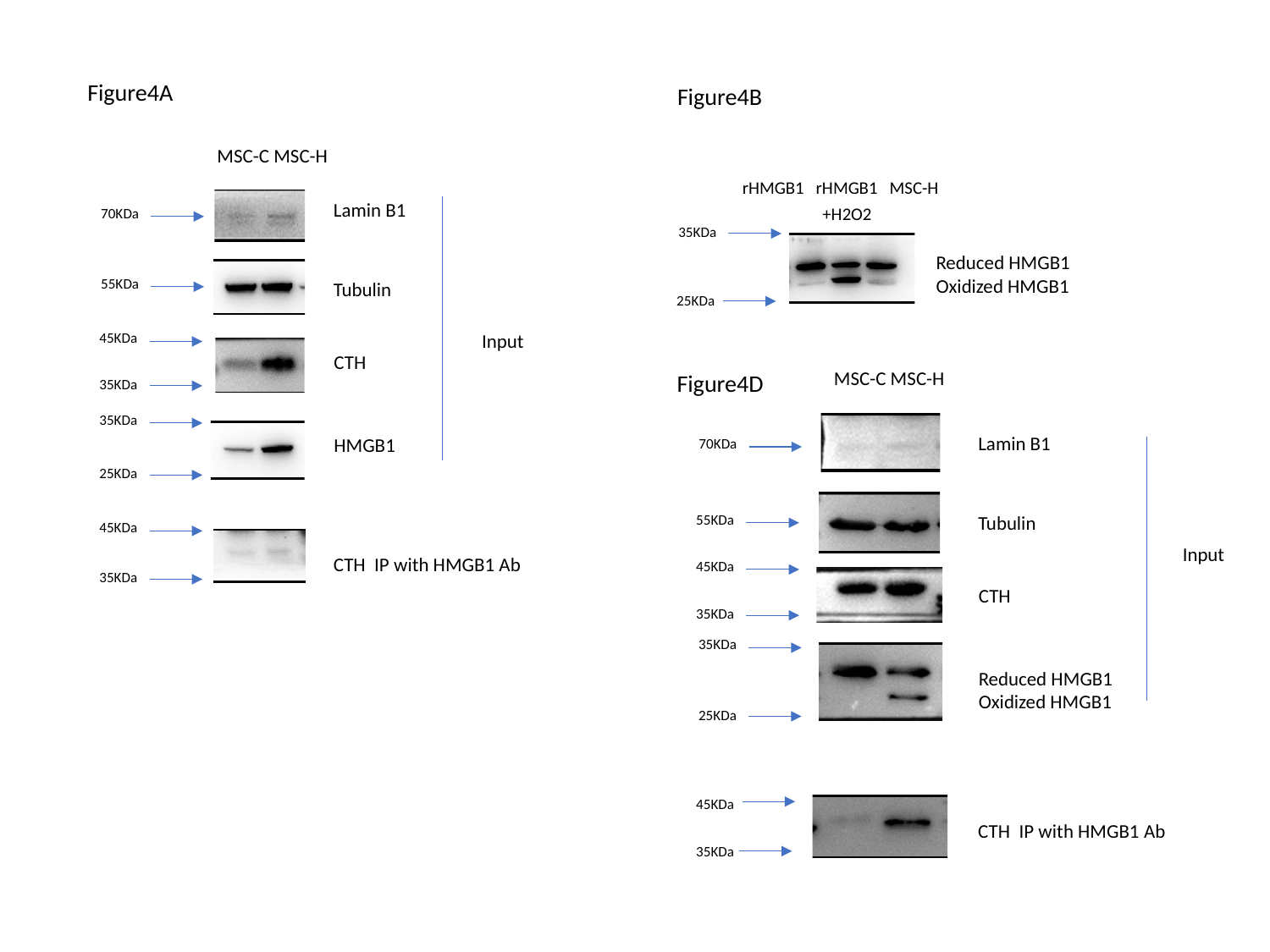

Figure4A
MSC-C MSC-H
Lamin B1
70KDa
55KDa
Tubulin
45KDa
Input
CTH
35KDa
35KDa
HMGB1
25KDa
45KDa
CTH IP with HMGB1 Ab
35KDa
Figure4B
rHMGB1 rHMGB1 MSC-H
+H2O2
35KDa
Reduced HMGB1
Oxidized HMGB1
25KDa
MSC-C MSC-H
Figure4D
Lamin B1
70KDa
55KDa
Tubulin
Input
45KDa
CTH
35KDa
35KDa
Reduced HMGB1
Oxidized HMGB1
25KDa
45KDa
CTH IP with HMGB1 Ab
35KDa

## Slide 6
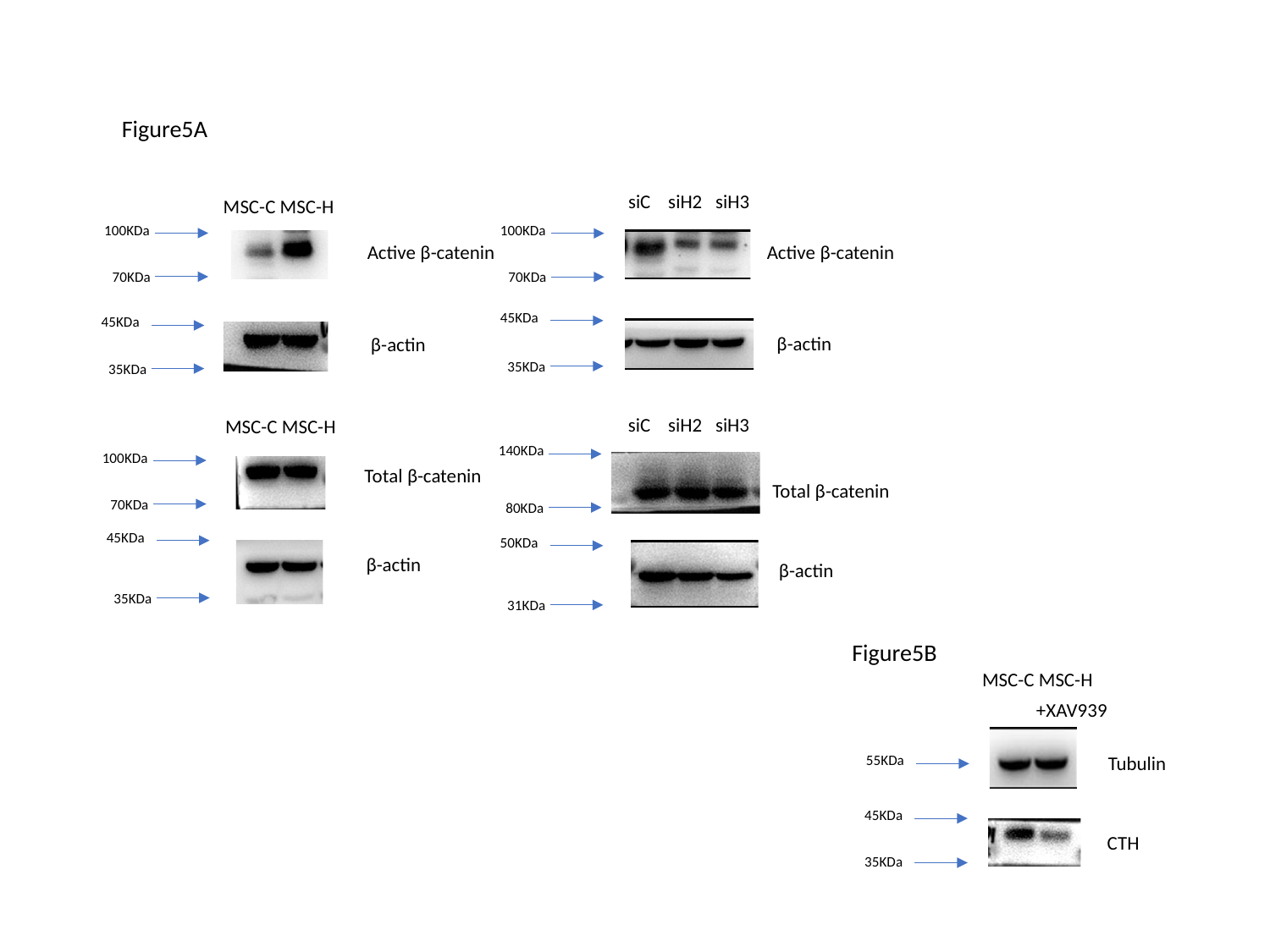

Figure5A
siC siH2 siH3
MSC-C MSC-H
100KDa
100KDa
Active β-catenin
Active β-catenin
70KDa
70KDa
45KDa
45KDa
 β-actin
 β-actin
35KDa
35KDa
siC siH2 siH3
MSC-C MSC-H
140KDa
100KDa
Total β-catenin
Total β-catenin
70KDa
80KDa
45KDa
50KDa
 β-actin
 β-actin
35KDa
31KDa
Figure5B
MSC-C MSC-H
+XAV939
55KDa
Tubulin
45KDa
CTH
35KDa

## Slide 7
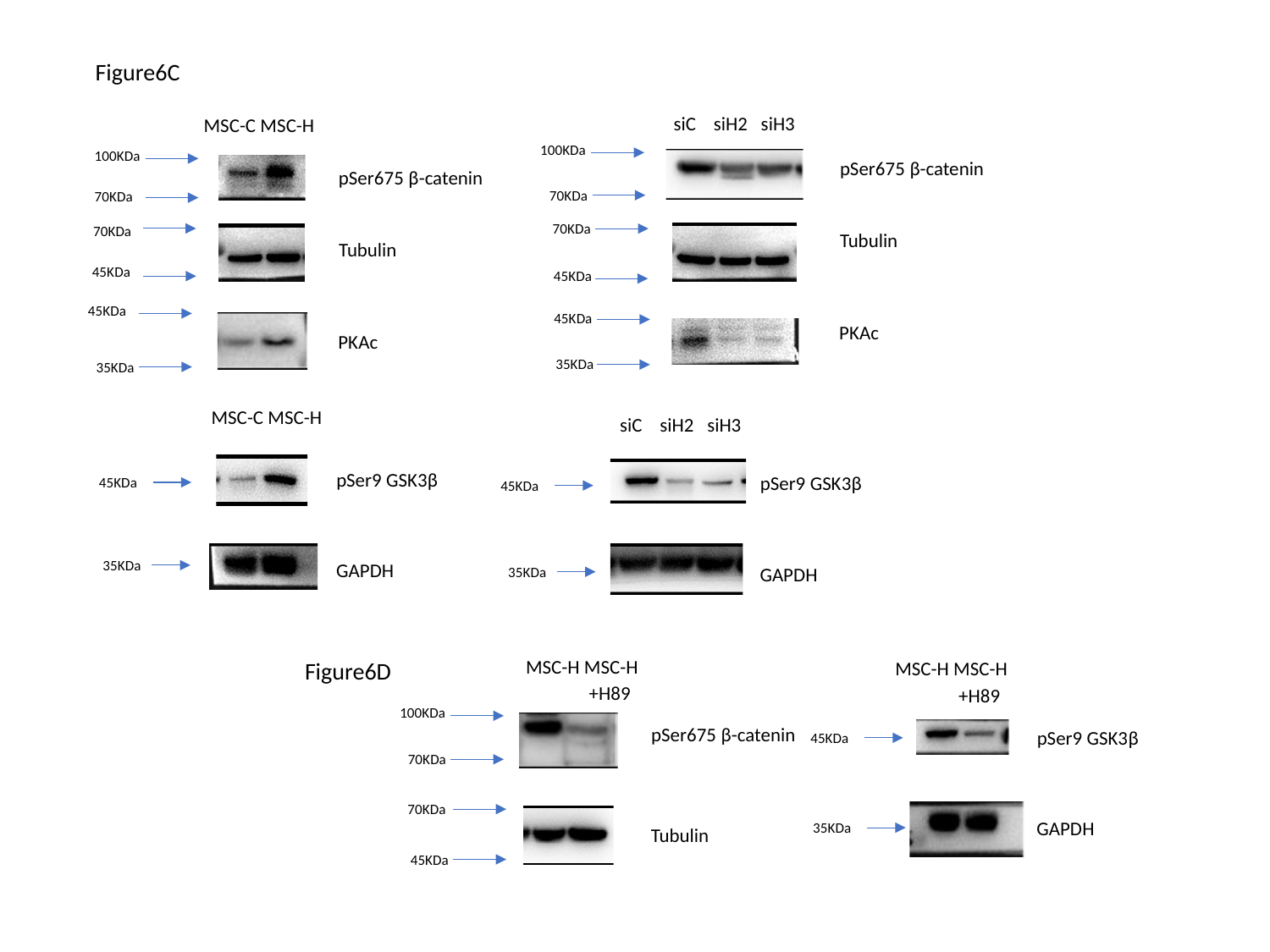

Figure6C
siC siH2 siH3
MSC-C MSC-H
100KDa
100KDa
pSer675 β-catenin
pSer675 β-catenin
70KDa
70KDa
70KDa
70KDa
Tubulin
Tubulin
45KDa
45KDa
45KDa
45KDa
PKAc
PKAc
35KDa
35KDa
MSC-C MSC-H
siC siH2 siH3
pSer9 GSK3β
pSer9 GSK3β
45KDa
45KDa
35KDa
GAPDH
GAPDH
35KDa
MSC-H MSC-H
Figure6D
MSC-H MSC-H
+H89
+H89
100KDa
pSer675 β-catenin
pSer9 GSK3β
45KDa
70KDa
70KDa
GAPDH
35KDa
Tubulin
45KDa
